# Genomic and Biological Characterization of a Broad-Host-Range KVP40-Like Bacteriophage vB_VpM-pA3B5 for Control of AHPND-Causing *Vibrio parahaemolyticus*

**DOI:** 10.4014/jmb.2602.02046

**Published:** 2026-07-02

**Authors:** Soojin Lim, Jee Eun Han, Seon Young Park, Tae Seon Cha, Keeman Lee, Hye Jin Jeon, Bumkeun Kim, So Young Bang, Yukyung Kim, Se Chang Park, Ji Hyung Kim

**Affiliations:** 1 Department of Food Science and Biotechnology, College of BioNano Technology, Gachon University, Seongnam 13120, Republic of Korea; 2 Laboratory of Aquatic Biomedicine, College of Veterinary Medicine and Research Institute for Veterinary Science, Seoul National University, Seoul 08826, Republic of Korea; 3 Institute for Veterinary Biomedical Science, Department of Veterinary Medicine, Kyungpook National University, Daegu 41566, Republic of Korea; 4 Department of Veterinary Medicine, Faculty of Veterinary Science, Chulalongkorn University, Bangkok 10330, Thailand; 5 Veterinary Drugs and Biologics Division, Animal and Plant Quarantine Agency, Gimcheon 39660, Republic of Korea

**Keywords:** Acute hepatopancreatic necrosis disease (AHPND), Jumbo bacteriophage, Biocontrol, Seafood safety

## Abstract

*Vibrio parahaemolyticus* harboring the pVA1 plasmid (*Vp_AHPND_*) causes acute hepatopancreatic necrosis disease (AHPND), a major threat to global shrimp aquaculture. Although bacteriophage (phage)-based biocontrol has emerged as a promising alternative to antimicrobial agents for controlling *Vp_AHPND_*, most studies have focused on disease prevention in culturing shrimp rather than post-harvest applications. In this study, a KVP40-like jumbo phage, designated vB_VpM-pA3B5, was isolated and characterized, and its biocontrol potential was evaluated in both *Vp_AHPND_*-contaminated shrimp products and shrimp bioassay. Phage vB_VpM-pA3B5 exhibited a broad host range, infecting globally distributed *Vp_AHPND_* strains, an AHPND-causing *V. campbellii* strain, and three additional *Vibrio* species. The phage demonstrated strong lytic activity, efficient replication, and high environmental stability. Genome analysis revealed a 243,570-bp linear dsDNA genome encoding 382 predicted ORFs and 28 tRNAs, with no virulence or antimicrobial resistance genes detected. Comparative genomic analysis indicated high synteny with the *Schizotequatrovirus* phages KVP40 and PVA8 and identified a unique tail fiber protein (ORF115) that may contribute to its broad host range. In *Vp_AHPND_*-contaminated shrimp products, the phage efficiently reduced bacterial loads and actively replicated during storage. In the shrimp bioassay, although cumulative mortality was not reduced, phage-treated shrimp exhibited alleviated hepatopancreatic lesions and lower *pirA* gene loads than the *Vp_AHPND_*-challenged group. These findings highlight the potential of phage vB_VpM-pA3B5 as a biocontrol agent for mitigating AHPND-associated risks in shrimp aquaculture and reducing the dissemination of *Vp_AHPND_* through shrimp products.

## Introduction

*Vibrio parahaemolyticus* is a widely distributed zoonotic pathogen in marine and estuarine environments and is a leading cause of seafood-borne gastroenteritis globally [1, 2]. In the global aquaculture industry, certain strains of *V. parahaemolyticus* (*Vp_AHPND_*) are responsible for acute hepatopancreatic necrosis disease (AHPND) in cultured shrimp, such as *Litopenaeus vannamei* and *Penaeus monodon*, which cause up to 100% mortality within 20–30 days post-stocking [3, 4]. Since its emergence in 2009, AHPND has spread across Asia and Latin America, challenging global shrimp production and food security [5, 6]. The virulence of *Vp_AHPND_* is attributed to the PirAB toxin encoded by the transferable pVA1 plasmid [7], which has been identified in other species of *Vibrio*, including *V. harveyi* and *V. campbellii* [8, 9]. Moreover, the extensive use of antibiotics in aquaculture has contributed to the emergence of multidrug-resistant strains, including *Vp_AHPND_*, thereby limiting the efficacy of traditional antibiotics and underscoring the need for alternative and sustainable approaches [10].

Beyond the economic losses in aquaculture, *Vp_AHPND_* may also have potential implications for seafood production and quality. Shrimp products are recognized as an important vehicle for seafood-borne illness worldwide [11-13], and the potential impact of shrimp pathogens on seafood safety has received increasing attention [14]. During AHPND infection, *Vp_AHPND_* colonization severely disrupts host microbial homeostasis, resulting in reduced microbial diversity and the overgrowth of *Vibrio* and *Photobacterium* spp. [15-17]. Such *Vibrio*-enriched environments, whether associated with primary *Vp_AHPND_* infection or secondary bacterial invasion [18], may facilitate the persistence and dissemination of bacterial populations carrying virulence factors or antimicrobial resistance determinants. Given the ecological significance of *Vibrio* spp. in aquaculture and seafood production environments [19], effective strategies to suppress *Vp_AHPND_* and limit its dissemination are needed to support sustainable shrimp production and improve seafood safety.

Bacteriophages (phages) could be a promising anti-Vibrio agent due to their high specificity, self-replicating nature, and minimal impact on the aquaculture environment or food quality [20]. Several *Vibrio*-specific phages, including A3S, Vpms, F8, F12, KVP40, and vB_Va_Val-yong3, have demonstrated biocontrol efficacy in aquaculture systems [21-24]. Moreover, other phages, including VPT02, BPVP-3325, and VVP001, have successfully reduced *Vibrio* contamination in seafood products such as oysters and ready-to-eat fish slices [25-27].

Among these, *Vibrio* phage KVP40 is one of the most well-characterized phages belonging to the genus *Schizotequatrovirus*, a group of jumbo myoviruses with large dsDNA genomes, a broad host range, and a strictly lytic replication cycle [28]. To date, at least 19 KVP40-like phages have been reported, sharing conserved genomic features and a broad host spectrum [20, 29, 30], suggesting their potential as biocontrol agents in aquaculture. Recent studies have identified several phages with lytic activity against *Vp_AHPND_* strains from different geographical regions, including podovirus-like (vB_VpaP_AL-1, VPK8), siphovirus-like (vB_VpaS_AL-2, pVp-1), and myovirus-like (vB_VpM-pA2SJ1, PVA8) phages [29, 31-34]. However, the biocontrol potential of KVP40-like phages against AHPND-causing *Vibrio* strains remains poorly understood. To date, phage PVA8 is the only KVP40-like phage evaluated against *Vp_AHPND_*. While PVA8 successfully reduced shrimp mortality in laboratory-scale trials, its host range evaluation was limited exclusively to Chinese isolates, highlighting the need for broader geographical sources [29]. Furthermore, although several KVP40-like phages have been identified, none have been investigated for their biocontrol efficacy in food models.

To address these limitations, we report a new KVP40-like *Schizotequatrovirus* phage, vB_VpM-pA3B5, with broad lytic activity against shrimp pathogenic *Vp_AHPND_* strains from multiple geographical origins. Based on comprehensive biological and genomic analyses, as well as the evaluation of biocontrol efficacy in *Vp_AHPND_*-contaminated shrimp products and shrimp bioassay, we propose vB_VpM-pA3B5 as a promising candidate for suppression of *Vp_AHPND_* and mitigation of its spread in seafood environments.

## Materials and Methods

### Bacterial Strains and Growth Conditions

A total of 21 strains of *Vibrio* spp. were used in this study, including five *Vp_AHPND_* strains (19-021-D1, 19-022-A1, CH49, 15-250/20, and 13-028/A3) and one AHPND-causing *V. campbellii* strain (16-904/1) isolated from various geographical regions, including South Korea, Thailand, Latin America, Vietnam, and Mexico. These strains included seven *V. parahaemolyticus*, two *V. harveyi*, two *V. campbellii*, two *V. alginolyticus*, three *V. mimicus*, two *V. splendidus*, and four other *Vibrio* species (*V. ulleungensis*, *V. orientalis*, *V. profundi*, and *V. vulnificus*). All bacterial strains were cultured in tryptic soy broth (TSB; BD Difco, USA) supplemented with 2.5% (w/v) NaCl (TSB+) and incubated at their respective optimal temperature: 25°C for *V. harveyi*, *V. campbellii*, *V. splendidus*, *V. orientalis*, and *V. vulnificus*; 30°C for *V. ullengensis* and *V. profundi*; and 37°C for all remaining strains, including the host strain *Vp_AHPND_* 13-028/A3. For long-term storage, all strains were preserved at -80°C in TSB+ containing 10% (v/v) glycerol.

### Phage Isolation and Propagation

Phage isolation was conducted as previously described [34], using *Vp_AHPND_* 13-028/A3 as the host. Seawater samples were collected from a seashore in Busan, South Korea, and stored at 4°C until use. Phage was directly enriched from the seawater sample without physical concentration to biologically amplify *Vp_AHPND_*-infecting phages. Briefly, 20 mL of a freshly prepared host culture (OD_600_ = 0.2) was mixed with 20 mL of seawater sample and incubated overnight at 37°C with shaking at 180 rpm. Following incubation, the mixture was centrifuged at 12,000 × *g* for 10 min (Beckman Coulter, USA), and the supernatant was filtered through a 0.45-μm polyvinylidene fluoride membrane filter (PVDF; Millipore, USA) to remove bacterial cells. Phages were confirmed using the double-layer agar (DLA) technique. Briefly, 6 mL of top agar (TSB+ containing 0.7% agar) inoculated with the host strain was overlaid onto a bottom agar plate (TSB+ containing 1.5% agar). After overnight incubation, a single clear plaque was selected, resuspended in 3% (w/v) NaCl-supplemented distilled water, serially diluted, and subjected to three consecutive rounds of single-plaque isolation to ensure genetic homogeneity. A high-titer phage lysate was subsequently prepared by infecting a log-phase host culture with a purified phage, followed by overnight incubation under the same conditions. The resulting culture was centrifuged and filtered to obtain a clear lysate, which was stored at 4°C for further characterization.

### Transmission Electron Microscopy

The morphology of the isolated phage was analyzed using transmission electron microscopy (TEM), as previously described [36]. A 10-μL aliquot of high-titer phage lysate (10^9^ PFU/mL) was applied to a 200-mesh Formvar/carbon-supported nickel grid (Ted Pella, USA) and allowed to adsorb for 5 min at room temperature. Excess liquid was removed using filter paper, and the grid was stained with 5% (w/v) uranyl acetate for 2 min. After staining, the grid was briefly rinsed with distilled water to remove the excess stain and air-dried for 1 h at room temperature. The grid was imaged using a transmission electron microscope (JEM-1011; JEOL Ltd., Japan) operating at an acceleration voltage of 80 kV.

### Host Range Determination

The host range of the isolated phages was determined by calculating the efficiency of plating (EOP) against 21 strains of *Vibrio* spp. as previously reported [37]. To prepare the bacterial lawn, 100 μL of the bacterial suspension was mixed with 6 mL of top agar and poured onto the bottom agar plate. Once solidified, 10 μL of serially diluted phage suspension (10^7^-10^2^ PFU/mL) was spotted on the surface of each lawn and incubated under optimal conditions. The EOP value was calculated as the ratio of the PFU/mL on the test strain to the PFU/mL on the primary host strain, *Vp_AHPND_* 13-028/A3. The infection efficiency was categorized into four levels: High (+++, EOP ≥ 0.5), Medium (++, 0.1 ≤ EOP < 0.5), Low (+, 0.001 ≤ EOP < 0.1), and Inefficient (-, EOP ≤ 0.001). Cases where a lysis zone was observed at high phage concentrations without individual plaque formation were defined as Lysis from without (LFW). All assays were performed in triplicate.

### Adsorption and One-Step Growth Curve

The replication dynamics of the phage were evaluated by measuring the adsorption rates and one-step growth curve, following a previously described method [27, 34] using the *Vp_AHPND_* strain 13-028/A3 as the host. For the adsorption assay, a log-phase host culture (10^7^ CFU/mL) was mixed with a phage suspension at a multiplicity of infection (MOI) of 0.01 and incubated at 37°C with shaking. Samples were collected at 0, 3, 5, 7, 10, 15, and 20 min post-infection and filtered through 0.45-μm PVDF membrane filters (Millipore) to remove adsorbed phages. The filtrates were serially diluted and titrated using DLA. The adsorption rate was calculated by comparing the phage titer remaining in the filtrate to the initial phage concentration. For the one-step growth curve analysis, a log-phase host culture (10^7^ CFU/mL) was infected with a phage solution at an MOI of 0.01 and incubated for 10 min to allow attachment to the host. This period was selected based on the adsorption assay, which showed approximately 84.9% phage attachment within 10 min. Following incubation, the mixture was centrifuged at 12,000 × *g* for 5 min (LaboGene, Denmark), and the pellet was resuspended in pre-warmed TSB+ to remove unattached phages. Two separate sets (1 mL each) of samples were collected at specific intervals (0, 3, 5, 7, 10, 15, 20, 25, 30, 35, and 40 min). To determine the eclipse period, one set of samples was treated with 10 μL of 100% chloroform (1% v/v final concentration; Samchun Chemical, Republic of Korea), vortexed for 30 s, and allowed to react at room temperature for 5 min to release intracellular phages. These samples were then filtered for titration. To determine the latent period and burst size, the other set of samples was immediately filtered through a PVDF membrane filter without chloroform treatment. All samples were titrated using the DLA method. The eclipse period was defined as the time elapsed prior to the first rise in phage titer in the chloroform-treated samples, and the latent period was determined as the time elapsed before the first increase in phage titer in the non-chloroform-treated samples. Finally, the burst size was calculated as the ratio of the net phage progeny produced at the plateau phase to the number of infected cells. The number of infected cells was estimated based on the initial host cell concentration, MOI, and the predetermined adsorption rate [38].

### Environmental Stability

Thermal, pH, and salinity stability tests of the isolated phages were performed as previously described [34] with minor modifications. For salinity stability, 100 μL of phage suspension was mixed with 900 μL of distilled water containing NaCl at final concentrations ranging from 0% to 5%. The mixtures were incubated at 4°C for 3 h, and the residual infectivity was determined using the DLA method. Based on these results, 3% NaCl solution was selected as the optimal diluent for subsequent thermal and pH stability tests. For thermal stability, 100 μL of the phage suspension was mixed with 900 μL of 3% NaCl solution and incubated at 4, 16, 25, 37, 45, 56, and 80°C for 3 h. Phage viability was evaluated using the DLA method. For pH stability, 100 μL of phage suspension was added to 900 μL of pH-adjusted buffer solutions (pH 2–10; Samchun Chemical, Republic of Korea), each supplemented with 3% NaCl. After incubation at 4°C for 3 h, residual infectivity was assessed. All experiments were conducted in triplicate, and statistical significance was evaluated by comparing the results with the control conditions (pH 7, 4°C, and 3% NaCl).

### Bacteriolytic Activity

The bacteriolytic activity of the phage against *Vp_AHPND_* 13-028/A3 was evaluated at various MOIs, following a previously described method [39] with minor modifications. Host cultures in the exponential growth phase (OD_600_ = 0.2) were independently inoculated with phage suspensions at MOIs of 0.01, 0.1, 1, and 10. The same amount of TSB+ was used as the positive control. All mixtures were then incubated at 37°C with shaking at 180 rpm for 8 h. Bacterial growth was monitored hourly by measuring the optical density at 600 nm (OD_600_) using a UV/Vis spectrophotometer (CAS, Republic of Korea). Each condition was tested in triplicate across three independent experiments.

### Genome Sequencing and Bioinformatics Analysis

Genomic DNA from the isolated phages was extracted using a QIAamp DNA kit (QIAGEN, Germany) according to the manufacturer’s instructions. The TruSeq DNA Nano Sample Preparation Kit (Illumina, USA) was used to prepare sequencing libraries, according to the manufacturer’s instructions. A total of 99,338 reads (14,987,776 total read bases, G+C content 42.4%, Q30:96.0%) were sequenced using the Illumina HiSeq platform (Illumina) and used for *de novo* assembly using SPAdes v3.13.0 software [40]. Genome annotation was performed using the Rapid Annotation Subsystem Technology server (https://rast.nmpdr.org/), and putative open reading frames (ORFs) and conserved domains were identified using BLASTp (https://blast.ncbi.nlm.nih.gov/Blast.cgi) and Interproscan v97.0 (https://www.ebi.ac.uk/interpro/) based on the Pfam v3.6 database (http://pfam.xfam.org/), respectively. Putative transmembrane and signal peptides were identified using TMHMM (version 2.0; https://services.healthtech.dtu.dk/services/TMHMM-2.0/) and SignalP (version 6.0; https://services.healthtech.dtu.dk/services/SignalP-6.0/). tRNAs were identified using tRNAscan-SE v.2.0 (http://lowelab.ucsc.edu/tRNAscanSE), and antibiotic resistance and virulence genes were screened using the Comprehensive Antibiotic Resistance Database (https://card.mcmaster.ca/) and the Virulence Factor Database (https://www.mgc.ac.cn/cgi-bin/VFs/v5/main.cgi), respectively. PhageTerm analysis was performed by mapping raw sequencing against the assembled genome to determine the physical structure and packaging mechanism [41]. The complete genome was visualized using the Proksee server (https://proksee.ca/) and displayed in a circular format to provide a comprehensive overview.

For phylogenetic analysis and comparative genome analysis, ViPTree v4.0 (https://www.genome.jp/viptree/) was used. A whole-genome tree was constructed using the genome sequences of eight *Schizotequatrovirus* phages (three KVP40-like phages, two ValKK3-like phages, one VH7D-like phage, and one unclassified phage), with *Vibrio* phage nt-1 (genus *Mylasvirus*) as an outgroup. Based on phylogenetic proximity, a comparative genome analysis was performed with two closely related members, KVP40 and PVA8, to assess their genomic similarities to the newly isolated phage. Also, VIRIDIC (http://viridic.icbm.de) was used to calculate the intergenomic similarities between the *Schizotequatrovirus* phages.

### Food Application

The biocontrol potential of the isolated phage was tested in shrimp powder as a food matrix. The experiment was designed to mimic sub-optimal conditions, such as improper refrigeration and thawing, or rehydration during the retail process. These conditions were selected as they provide a favorable environment for *Vibrio* contamination and rapid proliferation [42-44].

To prepare the food matrix, frozen blocks of *L. vannamei* (Pacific whiteleg shrimp) farmed in Gochang (Jeonbuk, Korea) were thawed at room temperature for 2 h, dried at 60°C for 18 h using a food dehydrator (L’EQUIP, Republic of Korea), and ground for 5 min using a food grinder (Shinil, Seoul, Korea). The powder was stored at -20°C until use and sterilized before the experiment by autoclaving at 121°C for 15 min, followed by overnight drying at 56°C. Three experimental groups were prepared: (i) positive control (*Vp_AHPND_* 13-028/A3 bacteria only), (ii) phage treatment group (both *Vp_AHPND_* 13-028/A3 and the phage), and (iii) negative control group (no inoculation with *Vp_AHPND_* 13-028/A3 and the phage). For both the positive control and phage treatment groups, 20 μL of *Vp_AHPND_* 13-028/A3 culture (approximately 10^9^ CFU/mL) was added to 5 g of sterilized powder mixed with 20 mL of TSB+. This setup was designed to reflect the aqueous environment of thawed or rehydrated shrimp products, which supports pathogen proliferation. In addition, the phage treatment group received 20 μL of phage lysate (10^9^ PFU/mL), resulting in an MOI of 1. All samples were incubated statically at 25°C to mimic room temperature food storage conditions. At 0, 3, 6, 9, and 12 h post-infection, 100 μL of each sample was collected and diluted with 900 μL of 0.1 M PBS buffer (pH 7.0), followed by centrifugation at 12000 g for 3 min (LaboGene). The supernatant was used for phage quantification using a spot assay on a DLA lawn. The pellet was washed twice with PBS, resuspended, and plated on thiosulfate citrate bile salt sucrose agar (TCBS; BD Difco, USA) for bacterial enumeration. The plates were incubated overnight at 37°C, and the typical green colonies of *V. parahaemolyticus* were counted. Phage counts were monitored only in the phage-treated group, whereas bacterial counts were assessed in all three groups.

### Shrimp Bioassay

The animal experiments were reviewed and approved by the Ethics and Welfare Committee of Kyungpook National University (Approval Numbers: KNU 2025-0188). All experimental procedures were performed in accordance with the relevant institutional and national guidelines and regulations.

For the shrimp bioassay, juvenile Pacific white shrimp (*P. vannamei*) were obtained from a commercial shrimp farm (Gochang-gun, Jeollabuk-do, Republic of Korea) and transported to the Laboratory of Aquatic Biomedicine, College of Veterinary Medicine, Kyungpook National University. Healthy shrimp (*N* = 18, mean body weight, 12.5 ± 0.5 g per shrimp) were acclimated before the experiment and randomly assigned to three treatment groups: (i) *Vp_AHPND_* strain 13-028/A3 (A3 [3]) challenge group (A3 group), (ii) A3 and phage challenged group (A3/phage group), and (iii) phage-only challenged group (phage group). Each treatment was conducted in duplicate using 20 L tanks containing 10 L of artificial seawater (25 ppt salinity) maintained at 28°C with continuous aeration. The A3 strain was cultured in TSB+ at 28°C with shaking at 200 rpm for 6 h until reaching approximately 2 × 10^9^ CFU/mL. For phage treatment, the A3 bacterial suspension was mixed with the phage suspension (approximately 2 × 10^9^ PFU/mL) at a 1:1 (v/v) ratio and maintained under conditions that permit phage adsorption before the experimental challenge. Shrimp in the A3 group were challenged by immersion with 10 mL of the bacterial suspension. Shrimp in the A3/phage group were exposed to 10 mL of the pre-incubated bacterial–phage mixture, whereas those in the phage group received 10 mL of the phage suspension only. Following the immersion challenge, shrimp were monitored every 12 h for 4 days, and cumulative mortality was recorded.

For histopathological examination, representative shrimp from each experimental group were sampled on days 1 and 4 post-challenge. The aseptically collected hepatopancreatic tissues were fixed in Davidson’s AFA fixative and processed according to standard histological procedures [45]. Histopathological changes were subsequently examined using a light microscope (Leica Microsystems, Germany). For quantitative PCR (qPCR) analysis, the hepatopancreatic tissues (approximately 30 mg) were aseptically collected from both dead and surviving shrimps, and total genomic DNA was extracted using the DNeasy^®^ Blood & Tissue Kit (QIAGEN, Germany) according to the manufacturer’s instructions. Then, the abundance of the AHPND toxin gene *pirA* was quantified by qPCR following the protocol described by Han et al [46].

### Statistical Analysis

All experiments were performed in triplicate (*n* = 3). Data are presented as mean ± standard deviation (SD). Statistical analyses were performed using GraphPad Prism 8 (GraphPad Software, USA). Statistical significance between the two groups was assessed using Student’s *t*-test. Asterisks indicate statistically significant differences (*, *p* < 0.05; **, *p* < 0.01; ***, *p* < 0.001; ****, *p* < 0.0001).

### Culture Deposition and Nucleotide Sequence Accession Numbers

The phage was deposited in the Korean Collection for Type Cultures (KCTC) under KCTC 16333BP, and the complete genome sequence was deposited in the GenBank database under accession number PV471468.

## Results

### Plaque and Virion Morphology of *Vibrio* phage vB_VpM-pA3B5

**Fig. 1:**
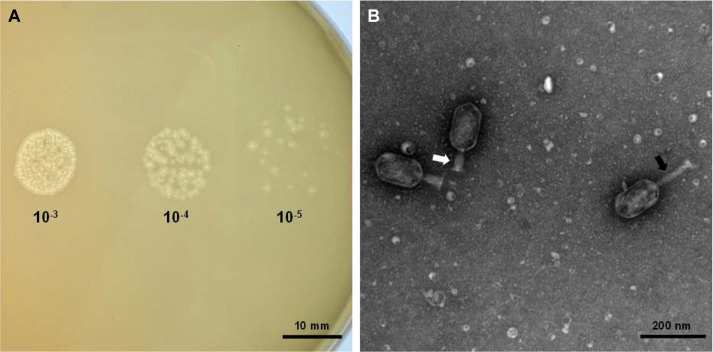
Morphological features of *Vibrio* phage vB_VpM-pA3B5. (**A**) Plaque formation on the *Vp_AHPND_* 13-028/A3 lawn following a spot assay with serial 10-fold dilutions of phage vB_VpM-pA3B5 (10^-3^, 10^-4^, and 10^-5^). Scale bar = 10 mm. (**B**) Transmission electron microscopy (TEM) image of phage vB_VpM-pA3B5. Black and white arrows indicate uncontracted and contracted tails, respectively. Scale bar = 200 nm.

A phage infecting *Vp_AHPND_* was isolated from seawater and produced clear plaques on a DLA lawn against its host *Vp_AHPND_* 13-028/A3 (Fig. 1A). TEM analyses revealed an elongated icosahedral capsid (88.1 ± 3.8 nm × 152.7 ± 3.2 nm) and a contractile tail (119.0 ± 2.7 nm extended; 53.6 ± 0.1 nm contracted) (Fig. 1B). The phage exhibited a myovirus-like morphology, was classified as belonging to the class *Caudoviricetes*, and was designated vB_VpM-pA3B5 following the standard viral nomenclature system [47].

### Host Range of *Vibrio* phage vB_VpM-pA3B5

The lytic efficiency of *Vibrio* phage vB_VpM-pA3B5 was evaluated by determining EOP values against 21 *Vibrio* strains (Table 1). The phage showed high infectivity (EOP ≥ 0.5) against *Vp_AHPND_* strains from Latin America (15-250/20), Vietnam (13-028/A3). Moderate infectivity (0.1 ≤ EOP < 0.5) was exhibited in AHPND-causing *V. campbellii* strain from Mexico (16-904/1), while low infectivity (0.001 ≤ EOP < 0.1) was observed in *Vp_AHPND_* strains from South Korea (19-021-D1 and 19-022-A1) and *V. orientalis* ATCC 33934. However, it was inefficient in *Vp_AHPND_* strain (CH49) from Thailand, with no plaque formation. Furthermore, while high-titer phage lysate produced lysis zones on *V. harveyi* ATCC 14126, *V. mimicus* ATCC 33653, and *V. splendidus* ATCC 33125 lawns, these strains were classified as LFW, as they failed to form individual plaques upon serial dilution.

### Biological Characteristics of *Vibrio* Phage vB_VpM-pA3B5

**Fig. 2:**
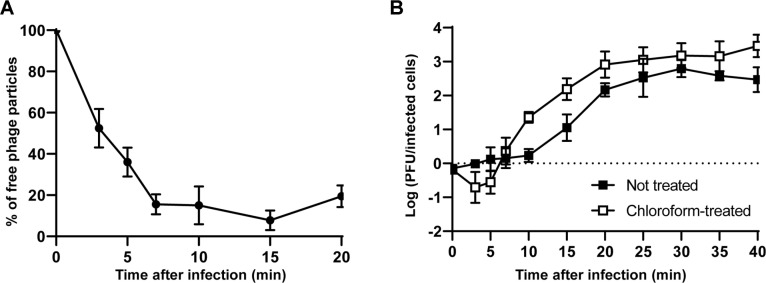
Replication dynamics of *Vibrio* phage vB_VpM-pA3B5 against *Vp_AHPND_* 13-028/A3 at a multiplicity of infection (MOI) of 0.01. (**A**) Adsorption kinetics of vB_VpM-pA3B5. (**B**) One-step growth curve of vB_VpM-pA3B5. Data are shown as mean SD from three independent experiments.

The biological characteristics of *Vibrio* phage vB_VpM-pA3B5 were investigated based on its replication efficiency and environmental stability against various stresses. The replication efficiency of the phage infecting *Vp_AHPND_* 13-028/A3 as the host strain was evaluated using adsorption kinetics and one-step growth curve analysis. In total, 84.9% of the free phages were adsorbed onto the host within 10 min (Fig. 2A). The one-step growth curve showed a short eclipse period (5 min) and latent period (10 min) with a burst size of 360.0 PFU/infected cell (Fig. 2B).

**Fig. 3:**
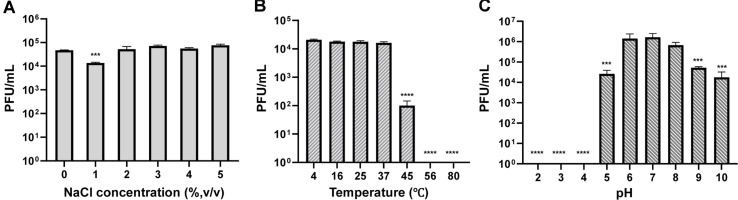
Environmental stability of *Vibrio* phage vB_VpM-pA3B5. Phage titers after exposure to varying (**A**) salinity, (**B**) thermal, and (**C**) pH conditions. Data are presented as mean SD from three independent replicates. Statistical significance relative to the control conditions (pH 7, 4 oC, and 3% NaCl) was determined using Student’s *t*-test and is indicated by asterisks (*p < 0.05, **p < 0.01, ***p < 0.001, ****p < 0.0001).

The environmental stability of the phages was evaluated under various salinity, pH, and temperature conditions. Under salinity stress, the phage showed a partial loss of infectivity in 1% NaCl solution (*p* < 0.001) (Fig. 3A). Under thermal stress, phages retained infectivity at temperatures ranging from 4°C to 37°C, but were unstable at temperatures above 45°C (*p* < 0.0001) (Fig. 3B). Under pH stress, the phage remained stable between pH 6 and 8. However, its infectivity declined below pH 5 and above pH 9 (*p* < 0.001), with complete inactivation under extremely acidic or alkaline conditions (*p* < 0.0001) (Fig. 3C).

### Bacteriolytic Activity of *Vibrio* Phage vB_VpM-pA3B5

**Fig. 4:**
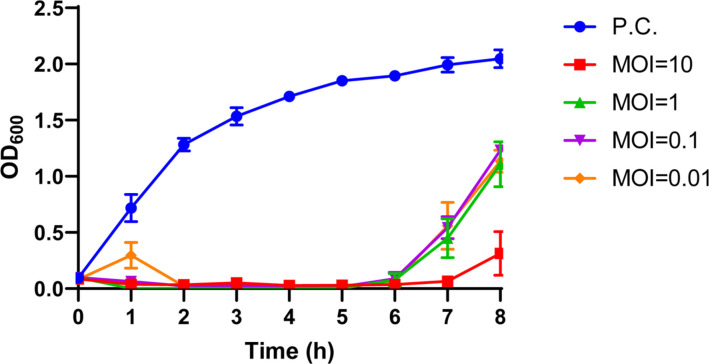
Bacteriolytic activity of *Vibrio* phage vB_VpM-pA3B5 against *Vp_AHPND_* 13-028/A3 at different multiplicities of infection (MOIs = 0.01, 0.1, 1, and 10). Data are expressed as the means SD of three independent experiments.

The bacteriolytic activity of the *Vibrio* phage vB_VpM-pA3B5 against *Vp_AHPND_* 13-028/A3 was assessed at MOIs of 0.01, 0.1, 1, and 10, and bacterial growth was monitored hourly for 8 h. In the uninfected control, the host bacteria grew rapidly, reaching an OD_600_ of 1.28 within 2 h of incubation. In contrast, phage treatment effectively suppressed bacterial growth for up to 6 h post-infection across all tested MOIs, even at the lowest MOI of 0.01. Although bacterial regrowth was observed between 6 and 8 h, treatment with the highest MOI (MOI = 10) effectively repressed this regrowth compared to the lower MOI treatments (Fig. 4).

### General Genomic Features of *Vibrio* Phage vB_VpM-pA3B5

**Fig. 5:**
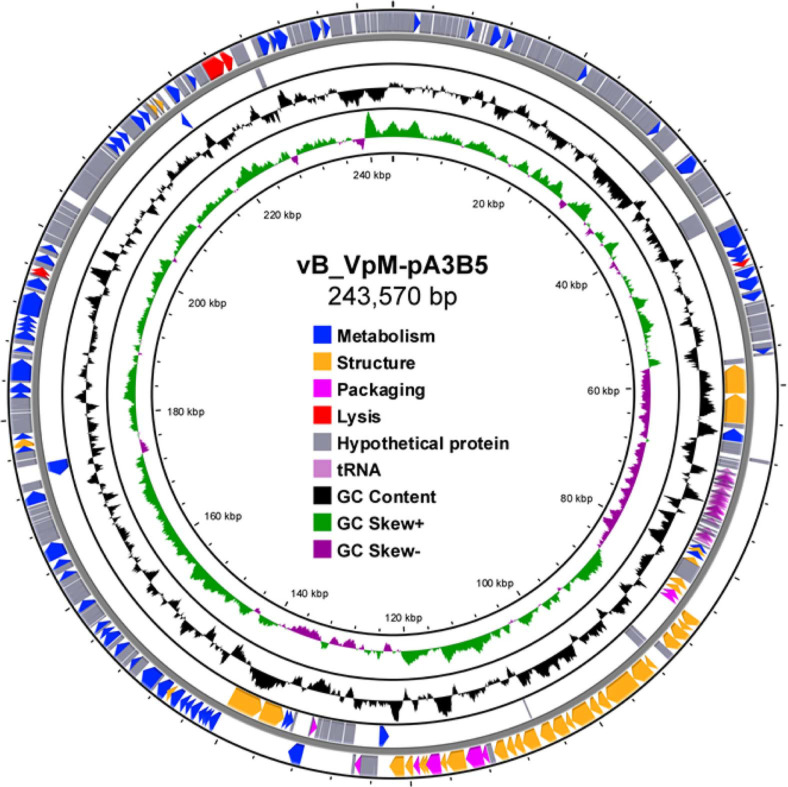
Genome map of *Vibrio* phage vB_VpM-pA3B5. The outer two rings show predicted ORFs encoded on the forward and reverse strands, respectively. ORFs are color-coded according to their putative functions: metabolism (blue), structure (yellow), packaging (pink), lysis (red), and hypothetical proteins (grey). The inner rings indicate GC content (black) and GC skew (green, positive values; purple, negative values). The genome map was generated using the Proksee server and is displayed as a circular representation for visualization purposes, although the phage genome is physically linear.

The complete genome of *Vibrio* phage vB_VpM-pA3B5 was sequenced using the Illumina HiSeq platform, and 99,338 reads were obtained with 61.4 coverage. The phage possesses a 243,570 bp sequence with a G+C content of 42.6%. Furthermore, PhageTerm analysis revealed a headful (*pac*) packaging mechanism, indicating that the genome is circularly permuted and terminally redundant. The genome contained 382 predicted ORFs with a coding density of 92.7% and 28 tRNA genes. No genes related to antibiotic resistance, virulence, or lysogenicity were identified. Functional annotation classified the 114 ORFs into four major categories: metabolism (67 ORFs), structure (34 ORFs), packaging (8 ORFs), and lysis (5 ORFs) (Fig. 5). Most of the predicted proteins shared a high amino acid identity (90.2–100%) with those of *Schizotequatrovirus* phages. The metabolism-related genes included those encoding DNA polymerase (ORF 282), helicases (ORFs 194 and 353), and DNA primase (ORF 220). The structural genes comprised head-associated (eight ORFs), neck (four ORFs), and tail-related proteins (22 ORFs). The tail genes were further categorized into sheath (ORFs 141, 169, 173, and 174), fiber (ORFs 115, 116, 139, 199, and 200), and baseplate components (ORFs 144, 145, 148–151, and 156–162). Genes involved in host recognition were identified, including the long-tail fiber proximal subunit (ORF 200), distal subunits (ORFs 115 and 116), and hinge connector protein (ORF 199). Packaging-related genes included the terminase small (ORF 171) and large (ORF 172) subunits, and lysis-associated genes included the transglycosylase SLT domain protein (ORF 96) and spanins (ORFs 286 and 287) (Supplementary Table S1).

### Comparative Genome Analysis of the *Vibrio* Phage vB_VpM-pA3B5

BLASTp analysis revealed that the terminase large subunit (*terL*) and major capsid protein (*MCP*) of *Vibrio* phage vB_VpM-pA3B5 shared >99% amino acid identity with those of *Vibrio* phage KVP40 (terL: AAQ64424; MCP: AAQ64432), indicating a close relationship with the genus *Schizotequatrovirus*.

**Fig. 6:**
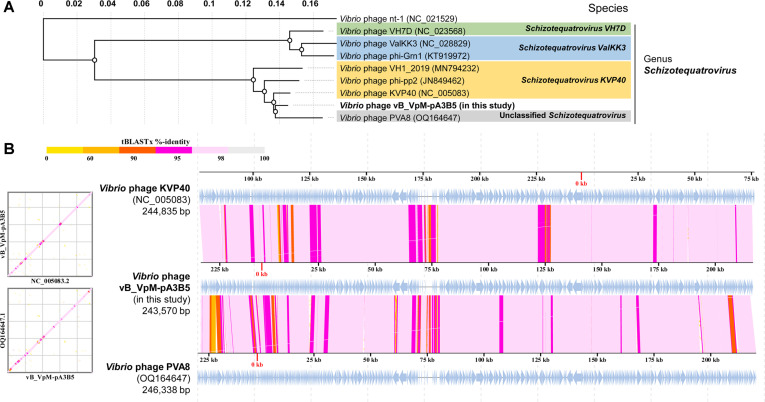
Whole-genome analysis of *Vibrio* phage vB_VpM-pA3B5 using VipTree. (**A**) Whole-genome-based phylogenetic tree constructed with seven *Schizotequatrovirus* phages: three KVP40-like phages (phi-pp2, VH1_2019, and KVP40), two ValKK3-like phages (ValKK3 and phi-Grn1), one VH7D-like phage (VH7D), and one unclassified *Schizotequatrovirus* (PVA8), with *Vibrio* phage nt-1 (*Myalvirus*) as an outgroup. (**B**) Genome comparison with closely related phages KVP40 and PVA8.

Based on these results, a whole-genome phylogenetic tree was constructed using seven *Schizotequatrovirus* phages: three KVP40-like phages (phi-pp2, VH1_2019, and KVP40), two ValKK3-like phages (ValKK3 and phi-Grn1), one VH7D-like phage (VH7D), and one unclassified *Schizotequatrovirus* (PVA8), with *Vibrio* phage nt-1 (*Myalvirus*) as an outgroup. The resultant phylogeny formed two major clusters: one with VH7D-like and ValKK3-like phages and the other with KVP40-like phages and PVA8. Phage vB_VpM-pA3B5 clustered closely with VH1_2019, phi-pp2, and KVP40 (Fig. 6A). Intergenomic similarities analyzed with *Schizotequatrovirus* phages showed that phage vB_VpM-pA3B5 shared >95% nucleotide identity with members of the species *Schizotequatrovirus* KVP40 (KVP40: 98.3%; phi-pp2: 96.4%), whereas it shares lower identity with other species (approximately 73.8%). Specifically, the intergenomic similarity values are 98.3% between pA3B5 and KVP40, and 96.2% between pA3B5 and PVA8 (Fig. S1).

Whole-genome alignment of KVP40 and PVA8 revealed that these phages shared over 95% tBLASTx identity across their genomes with conserved gene synteny, indicating a high level of overall similarity (Fig. 6B). To explore the potential genetic factors underlying host range differences, we compared the long tail fiber (LTF) proximal (ORF 200, 3771 bp) and distal subunit genes (ORF 115, 3258 bp; ORF 116, 3276 bp) of vB_VpM-pA3B5 with their homologs in KVP40 and PVA8, respectively. The proximal subunit of LTF showed high conservation, sharing 99.84% amino acid identity with KVP40 and 99.52% with PVA8. Among the distal subunit genes, ORFs 115 and 116 were almost identical to those of KVP40, with 99.8% identity and 100% sequence coverage. With PVA8, ORF 115 exhibited 95.8% identity with 74% coverage, whereas ORF 116 retained 98.4% identity with full coverage.

### Biocontrol Efficacy of *Vibrio* Phage vB_VpM-pA3B5 in Shrimp Powder

**Fig. 7:**
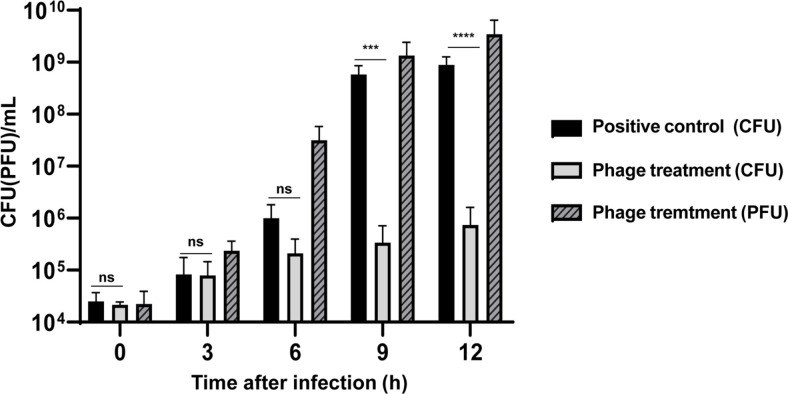
Biocontrol efficacy of *Vibrio* phage vB_VpM-pA3B5 in dried shrimp powder artificially contaminated with *Vp_AHPND_* 13-028/A3. Samples were divided into a positive control (bacteria only) and phage treatment (bacteria and phage, MOI = 1) and incubated statically at 25°C. Bacterial counts (CFU) were assessed at 0, 3, 6, 9, and 12 h post-infection, while phage titers (PFU) were measured in the phage-treated group. Data are presented as mean ± SD from three independent replicates. Statistical significance in CFU between control and phage-treated groups at each time point was assessed using Student’s *t*-test and is indicated by an asterisk (ns, not significant; *, *p* < 0.05, **, *p* < 0.01; ***, *p* < 0.001; ****, *p* < 0.0001).

The biocontrol potential of vB_VpM-pA3B5 against *Vp_AHPND_* 13-028/A3 was evaluated using *Vp_AHPND_*-contaminated shrimp powder at an MOI of 1. Viable bacterial counts and phage titers were monitored at 0, 3, 6, 9, and 12 h post-infection. In the (i) positive control, bacterial loads increased markedly after 6 h and peaked at 12 h, confirming that *Vp_AHPND_* readily proliferates in the shrimp powder matrix. In the (ii) phage-treated group, while no significant differences in bacterial counts were observed during the first 6 h compared to the control, the phage treatment subsequently exerted a significant inhibitory effect. Specifically, it achieved maximum reductions of 3.2 log CFU/mL at 9 h and 3.0 log CFU/mL at 12 h post-infection compared to the positive control. Although the treatment did not completely suppress bacterial growth over the entire 12 h period, it significantly restricted bacterial proliferation. At the same time, the phage titers increased steadily (Fig. 7). No bacterial growth was observed in the group (iii) at any time point (data not shown).

### Protective Effects of *Vibrio* Phage vB_VpM-pA3B5 in the Shrimp Bioassay

No differences in cumulative mortality were observed between the A3 and A3/phage groups during the experimental period, with both groups exhibiting a mortality rate of 33.3%. In contrast, no mortality was recorded in the phage group (Data not shown).

**Fig. 8:**
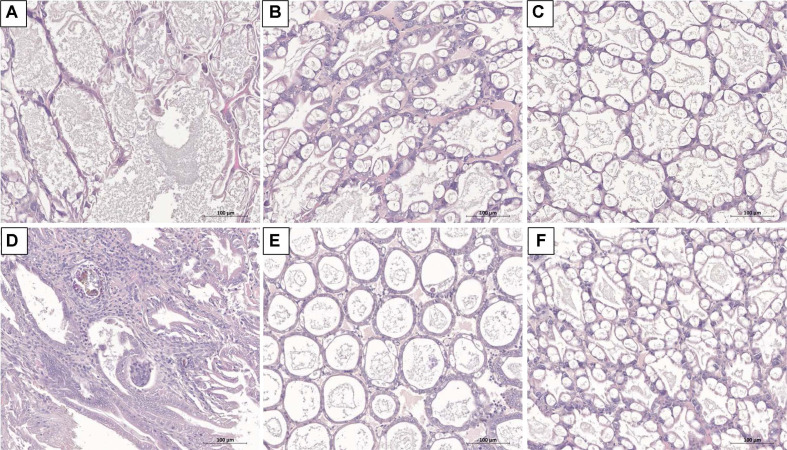
Histopathological analysis of the shrimp hepatopancreas following challenge with *Vp_AHPND_* 13-028/A3 and phage vB_VpM-pA3B5. Representative hepatopancreatic sections collected at day 1 post-challenge are shown for the A3 group (**A**) A3/phage group (**B**) and phage group (**C**). Representative sections collected at day 4 post-challenge are shown for the A3 group (**D**) A3/phage group (**E**) and phage group (**F**). Hepatopancreatic tissues were stained with hematoxylin and eosin (H&E) and examined by light microscopy. Scale bars = 100 μm.

Despite the absence of differences in mortality, histopathological examination revealed marked differences in hepatopancreatic lesions between the A3 and A3/phage groups. At day 1 post-challenge, shrimp in the A3 group exhibited severe AHPND-associated lesions, including extensive sloughing of tubular epithelial cells into the lumen and the disappearance of lipid droplets as well as hepatopancreatic R and B cells (Fig. 8A). In contrast, the A3/phage group showed no apparent epithelial sloughing, and the tubular structure, together with hepatopancreatic R and B cells, remained largely preserved (Fig. 8B). No histopathological abnormalities were observed in the phage group (Fig. 8C). At day 4 post-challenge, severe tissue damage persisted in the A3 group, characterized by extensive epithelial sloughing and hemocyte infiltration within the hepatopancreas (Fig. 8D). Although tubule atrophy and a reduction in hepatopancreatic R and B cells were observed in the A3/phage group, epithelial sloughing was absent, and the overall tissue architecture was considerably less affected than that observed in the A3 group (Fig. 8E). The phage group maintained normal hepatopancreas morphology throughout the experiment (Fig. 8F).

The qPCR targeting the *pirA* toxin gene further demonstrated the effect of phage treatment on AHPND progression. In dead shrimp, *pirA* copy numbers ranged from 3.15 × 10^6^ to 4.33 × 10^6^ copies/μL in the A3 group, whereas substantially lower copy numbers, ranging from 2.15 × 10^5^ to 4.90 × 10^5^ copies/μL, were detected in the A3/phage group. In surviving shrimp collected at the end of the experiment, *pirA* copy numbers ranged from 4.14 × 10^3^ to 1.42 × 10^4^ copies/μL in the A3 group and from 5.65 × 10^3^ to 1.12 × 10^4^ copies/μL in the A3/phage group (Table S2).

## Discussion

AHPND, primarily caused by *V. parahaemolyticus* carrying plasmid-borne PirAB toxins, is a major threat to global shrimp aquaculture [3, 48]. With growing concerns regarding antibiotic resistance, bacteriophages have emerged as promising alternatives for controlling *Vp_AHPND_*.

Several phages in the class *Caudoviricetes* have shown notable biocontrol potential. For instance, the unclassified phage vB_VpM-pA2SJ1 and *Malculvirus* phage vB_VpaP_AL-1 (family *Autographiviridae*) exhibit lytic activity against Asian and Mexican *Vp_AHPND_* strains, respectively, and have well-characterized biological and genomic profiles [31, 34]. Similarly, the *Vipnavirus* phage pVp-1 (family *Demerecviridae*) lysed Asian and Mexican strains and improved shrimp survival under *in vivo* AHPND challenge [33]. In addition, *Schizotequatrovirus* phage PVA8 (family *Straboviridae*), which is closely related to Jumbo phage KVP40, has a broad host range across *Vibrio* species, including Chinese *Vp_AHPND_* strains. PVA8 treatment reduced shrimp mortality in AHPND challenge assays and lowered *Vibrio* concentrations in commercial shrimp production environments [29]. While these studies highlight the potential of phage biocontrol against AHPND, most have focused on live shrimp, with limited attention paid to food safety and post-harvest applications.

In this study, we isolated the *Vp_AHPND_*-infecting jumbo phage, vB_VpM-pA3B5, from seawater in Korea. Based on morphological and genetic analyses, the isolated phage revealed a myovirus-like morphology with an elongated capsid (Fig. 1) and a 243,570 bp linear dsDNA genome (G + C content, 42.6%), consistent with previously described jumbo *Vibrio* phages [49-51]. The sequenced genome of vB_VpM-pA3B5 was similar to that of *Vibrio* phage KVP40, which is one of the most well-characterized representative phages of the genus *Schizotequatrovirus*. Whole-genome-based phylogenetic analyses revealed that phage vB_VpM-pA3B5 clustered well with the KVP40-like clade, showing high similarity to phages KVP40 and PVA8 [28, 29] (Fig. 6A). Intergenomic similarity analysis further supports taxonomical classification. As vB_VpM-pA3B5 shared >95% similarity with every phage within the species *Schizotequatrovirus* KVP40 (KVP40: 98.3%; phi-pp2: 96.4%, VH1_2019: 95.1%), it is classified as a new isolate of this species. Specifically, pA3B5 showed high identity with KVP40 (98.3%) and unclassified *Schizotequatrovirus* phage PVA8 (96.2%). These findings align with the whole-genome phylogenetic analysis, which clustered pA3B5 and PVA8 closely within the *Schizotequatrovirus* *KVP40* clade, implying the need to update the current taxonomic classification (Fig. S1).

Moreover, genome alignment between phages vB_VpM-pA3B5 and KVP40 revealed a conserved gene organization associated with metabolism, genome packaging, host cell lysis, and virion morphogenesis. Given that KVP40 is a well-characterized broad-host-range lytic phage [28,52], the high genomic similarity between KVP40 and vB_VpM-pA3B5 may explain the broad host range and strong lytic activity observed for vB_VpM-pA3B5 in this study. Additional genome analysis confirmed the absence of genes associated with lysogeny, virulence, and antibiotic resistance, supporting its safety for application. However, vB_VpM-pA3B5 showed different or advantageous biological characteristics compared to phages KVP40 and PVA8, particularly in terms of bacterial infectivity. First, phage vB_VpM-pA3B5 demonstrated a distinct host spectrum in several *Vibrio* species, as it can infect and reproduce in *V. campbellii* and *V. orientalis* compared to phages KVP40 and PVA8 [29, 48] (Table 1). Second, while the efficacy of PVA8 was validated exclusively against Chinese isolates [29], vB_VpM-pA3B5 proved effective against a geographically diverse array of *Vp_AHPND_* strains originating from South Korea, Vietnam, and Latin America. Third, the phage showed antibacterial activity against *V. campbellii* and *V. harveyi*. Although the interaction with *V. harveyi* was characterized as lysis from without (LFW), the observed lysis in high titer is noteworthy. Given that *V. harveyi* and *V. campbellii* are recognized as recipients of the *PirAB*-carrying pVA1 plasmid [3, 6], the lytic activity of vB_VpM-pA3B5 against these species suggests its potential in mitigating the environmental dissemination of AHPND, though further inhibition studies are warranted. Comparative analysis of LTF proteins between the phages showed that while the proximal LTF subunit (ORF 200) was highly conserved, the distal subunits (ORFs 115 and 116) displayed sequence divergence, particularly in ORF 115, compared to their homologs in PVA8. KVP40 and PVA8 have been reported to lack infectivity against both *V. campbellii* and *V. harveyi*, although vB_VpM-pA3B5 successfully infected *V. campbellii*. Although ORF 115 showed sequence divergence relative to PVA8, its high similarity to KVP40 suggests that tail fiber sequence alone is insufficient to explain the observed host range differences [53, 54]. Additional determinants of host specificity warrant further investigation. The unique infectivity of vB_VpM-pA3B5 strongly supports its potential application in controlling *Vp_AHPND_* in shrimp aquaculture.

One-step growth curve analysis revealed that *Vibrio* phage vB_VpM-pA3B5 possesses a short eclipse period (5 min) and latent period (10 min), coupled with a large burst size of 360.0 PFU/infected cell (Fig. 2). This latent period is among the shortest reported for KVP40-like phages, which typically range from *V. anguillarum* phage PVA23 (10 min) [24] to *V. alginolyticus* phage Va3 (150 min) [55]. Despite their genetic proximity, the factor determining the latent period requires further confirmation; however, it is hypothesized that this may be linked to the metabolic state and replicational characteristics of their respective hosts [42, 56], consistent with the broad host range and host diversity characteristic of the KVP40-like phages [57]. Furthermore, these replication parameters surpass those of other *Vp_AHPND_*-infecting phages, such as vB_VpM-pA2SJ1 [34], vB_VpaP_AL-1 [31], and PVA8 [29], which exhibit longer latent periods (10–25 min) and smaller burst sizes (85–309 PFU/ infected cell). Consistent with these robust replication kinetics, the phage exhibited potent bacteriolytic activity *in vitro*, effectively suppressing host growth at a remarkably low MOI of 0.01 with an efficacy comparable to that at an MOI of 10 (Fig. 4). However, prolonged incubation eventually led to bacterial regrowth, suggesting the possible emergence of phage-insensitive populations. This phenomenon, which has been observed with several other *Vp_AHPND_* phages, is a common evolutionary consequence of phage-host interactions under strong selective pressure, often associated with the modification or loss of bacterial surface receptors [58, 59]. The phage maintained stability across pH ranges of 6–8 and temperatures from 4 to 37°C. Regarding salinity, the phage remained stable at 0% and 2-5% NaCl solution, although a minor reduction in infectivity was observed after exposure to 1% NaCl solution (Fig. 3). While marine phages typically require ions for structural stability [60], several studies have reported exceptions where virion stability is maintained at low salinities (0–1.5%) regardless of host requirements [61-63]. Consistent with these reports, vB_VpM-pA3B5 exhibited stability even at 0% NaCl solution, suggesting that it is well-adapted to survive in a coastal environment subjected to significant salinity fluctuation [64]. These results support the potential of vB_VpM-pA3B5 as an effective biocontrol agent in food environments based on its strong lytic activity, robust replication dynamics, and stability under varied environmental conditions.

The ability of *V. parahaemolyticus* to survive common seafood processing conditions, such as freezing and drying [65], has raised concerns about the persistence of *Vp_AHPND_* in shrimp products [66]. While bacterial growth is generally restricted under proper dry or frozen storage conditions, these pathogens can persist and subsequently recover and proliferate when the product is thawed, rehydrated, or subjected to temperature abuse during retail [42-44]. This risk is highly relevant to dried shrimp due to typical room-temperature storage and consumption without reheating [65-68]. Furthermore, effective phage biocontrol inherently requires an aqueous environment for a metabolically active host [56, 69]. Therefore, rather than presenting a general storage phase, we utilized wet shrimp paste as an alternative food contamination model [70, 71]. Under these conditions, phage vB_VpM-pA3B5 proved its potential efficacy against *Vp_AHPND_*-contaminated shrimp products, significantly reducing bacterial load by > 3 log CFU within 12 h post-infection (Fig. 7). This reduction was accompanied by an increase in the number of phage virions, thus indicating active replication. Compared to other *Vibrio* phages used for the decontamination of *V. parahaemolyticus* in seafood, such as VVP001 (MOI = 10^5^) [27], BPVP-3325 (MOI = 10^2^) [26], and VPT02 (MOI = 1) [25], phage vB_VpM-pA3B5 demonstrated biocontrol efficacy at an MOI of 1, supporting its potential for food safety application. The shrimp bioassay provided additional evidence supporting the biocontrol potential of phage vB_VpM-pA3B5 against *Vp_AHPND_*. Although phage application did not reduce cumulative mortality under the experimental conditions tested, phage-treated shrimp exhibited markedly alleviated hepatopancreatic lesions, including reduced epithelial sloughing and improved preservation of hepatopancreatic architecture compared with untreated infected shrimp. Furthermore, lower *pirA* toxin gene loads were detected in phage-treated shrimp, suggesting that phage-mediated suppression of *Vp_AHPND_* may reduce toxin-associated tissue damage even when a measurable survival benefit is not observed. These findings complement the food decontamination results and further support the potential application of phage vB_VpM-pA3B5 as a biocontrol agent for reducing both *Vp_AHPND_* contamination in shrimp products and AHPND-associated pathological impacts in shrimp.

This study provides a comprehensive characterization of the KVP40-like *Vibrio* phage vB_VpM-pA3B5, which exhibited a broad host range against AHPND-causing and other *Vibrio* species. In a shrimp powder model, phage treatment significantly reduced *Vp_AHPND_* loads in an artificially contaminated food matrix. Although cumulative mortality was not reduced in the shrimp bioassay, phage treatment alleviated AHPND-associated histopathological lesions and reduced *pirA* toxin gene loads in infected shrimp. Together, these findings expand the potential application of phage-based biocontrol from disease management in shrimp aquaculture to the post-harvest control of AHPND-causing *Vibrio*s in seafood products.

## Supplemental Materials

Supplementary data for this paper are available on-line only at http://jmb.or.kr.



## Figures and Tables

**Table 1:** Infectivity of *Vibrio* phage vB_VpM-pA3B5 against *Vibrio* strains used in this study and their detailed information.

Bacterial strains	Geographical origin	Isolation source	EOP value^a^	Infectivity^b^	Reference
*V. parahaemolyticus*					
19-021-D1^d^	South Korea	Shrimp	0.01	+	[35]
19-022-A1^d^	South Korea	Shrimp	0.06	+	[35]
CH49^d^	Thailand	Shrimp	0	-	[35]
15-250/20^d^	Latin America	Shrimp	0.56	+++	[35]
13-028/A3^d^	Vietnam	Shrimp	1	+++	[35]
ATCC 17802^T^	Japan	Clinical	6.17	+++	ATCC
ATCC 33844	Japan	Clinical	2.09	+++	ATCC
*V. harveyi*					
LB4	USA	Shrimp	0	-	[35]
ATCC 14126^T^	USA	Marine animal	0	LFW^c^	ATCC
*V. campbellii*					
16-904/1^d^	Mexico	Shrimp	0.33	++	[3]
ATCC 25920^T^	USA	Seawater	0	-	ATCC
*V. alginolyticus*					
KACC 14902	South Korea	Environment	0	-	KACC
ATCC 17749^T^	Japan	Clinical	0.9	+++	ATCC
*V. mimicus*					
A-1	South Korea	Not specified	0	-	Lab collection
gyoB	South Korea	Not specified	0.46	++	Lab collection
ATCC 33653^T^	USA	Clinical	0	LFW^c^	ATCC
*V. splendidus*					
KACC 18110	South Korea	Marine animal	0	-	KACC
ATCC 33125^T^	Not specified	Marine animal	0	LFW^c^	ATCC
Other *Vibrio* spp.					
*V. ulleungensis* KACC 22258^T^	South Korea	Marine animal	0	-	KACC
*V. orientalis* ATCC 33934^T^	China	Environment	0.06	+	ATCC
*V. profundi* KACC 18555^T^	China	Environment	0	-	KACC
*V. vulnificus* KCTC 8599	South Korea	Eel	0	-	KCTC

^a^ The efficiency of plating (EOP) was determined by dividing the phage titer (PFU/mL) obtained on each test strain by the corresponding phage titer obtained on the reference host strain, *Vp_AHPND_* 13-028/A3.

^b^ Infectivity was categorized as: +++ (high, EOP ≥ 0.5); ++ (moderate, 0.1 ≤ EOP < 0.5); + (low, 0.001 ≤ EOP < 0.1); and - (inefficient, EOP ≤ 0.001)

^c^ LFW (lysis from without): formation of a clear zone at high phage concentration without individual plaque formation.

^d^ AHPND-causing *Vibrio* strains.
