# Supplementary material for: Genomic and Biological Characterization of a Broad-Host-Range KVP40-Like Bacteriophage vB_VpM-pA3B5 for Control of AHPND-Causing Vibrio parahaemolyticus
Source: J Microbiol Biotechnol. 2026 Jul 2;36:e2602046. doi: 10.4014/jmb.2602.02046 (PMC13373721; doi:10.4014/jmb.2602.02046)
Supplement: Supplementary file 1 [file jmb-36-e2602046-supple.pdf]

J. Microbiol. Biotechnol. 2026. 36: e2602046

https://doi.org/10.4014/jmb.2602.02046

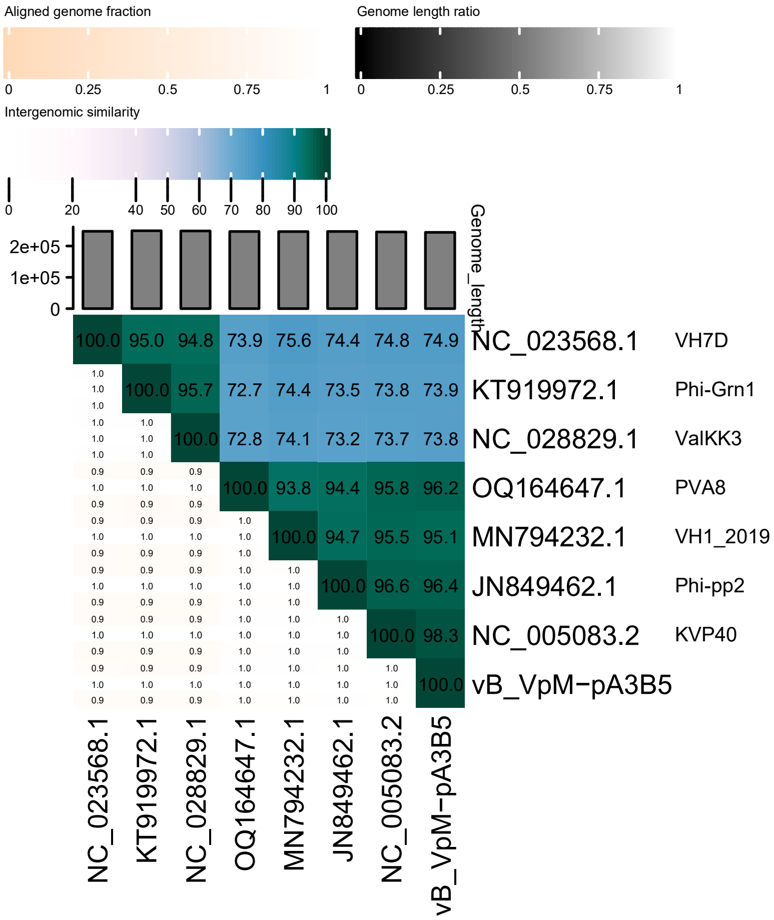

Fig. S1. Intergenomic similarity analysis of *Vibrio* phage vB_VpM_pA3B5 with seven *Schizotequatrovirus* phages was calculated using VIRIDIC.

Table S1. Features of the predicted open reading frames (ORFs) of *Vibrio* phage vB_VpM-pA3B5 and its homology against other phages available in the Genbank database. Putative functions and conserved domains were predicted with BLASTp and InterProscan, respectively.

Group	Gene product				Putative function [Conserved domain]	Best match virus (e-value)	Identity (%)	TMHHM	SignalP
	ORF No.	range	strand	length (aa)					
	1	2 - 208	+	68	Hypothetical protein	*Vibrio* phage KVP40 (4e-37)	100	0	N
	2	201 - 521	+	106	Hypothetical protein	*Vibrio* phage V09 (3e-74)	100	0	N
	3	518 - 952	+	144	Hypothetical protein	*Vibrio* phage KVP40 (6e-104)	100	0	N
	4	949 - 1134	+	61	Hypothetical protein	*Vibrio* phage KVP40 (2e-38)	100	0	N
	5	1131 - 1286	+	51	Hypothetical protein	*Vibrio* phage V09 (3e-30)	100	0	N
	6	1283 - 1750	+	155	Hypothetical protein	*Vibrio* phage V09 (5e-109)	98.7	0	N
	7	1747 - 2073	+	108	Hypothetical protein	*Vibrio* phage V09 (9e-75)	100	0	N
	8	2070 - 2243	+	57	Hypothetical protein	*Vibrio* phage V05 (4e-32)	98.3	2	N
Metabolism	9	2243 - 2827	+	194	Thymidine kinase [IPR001267; Thymidine_kinase; Thymidine kinase]	*Vibrio* phage VH1_2019 (3e-144)	99.5	0	N
	10	2811 - 3113	+	100	Hypothetical protein	*Vibrio* phage VH1_2019 (2e-69)	100	0	N
	11	3110 - 3460	+	116	Hypothetical protein	*Vibrio* phage KVP40 (6e-80)	100	0	N
	12	3457 - 3780	+	107	Hypothetical protein	*Vibrio* phage KVP40 (9e-75)	100	0	N
	13	3780 - 4073	+	97	Hypothetical protein	*Vibrio* phage KVP40 (4e-65)	100	0	
	14	4210 - 4809	+	199	Hypothetical protein	*Vibrio* phage V09 (6e-143)	100	0	N
	15	4809 - 4979	+	56	Hypothetical protein	*Vibrio* phage KVP40 (6e-33)	100	0	N
	16	4989 - 5549	+	186	Hypothetical protein	*Vibrio* phage KVP40 (5e-137)	100	0	N
	17	5582 - 6139	+	185	Hypothetical protein	*Vibrio* phage KVP40 (3e-134)	100	0	N
	18	6186 - 6779	+	197	Hypothetical protein	*Vibrio* phage phi-pp2 (2e-148)	100	0	N
	19	6851 - 7093	+	80	Hypothetical protein	*Vibrio* phage phi-pp2 (3e-51)	100	0	N
	20	7152 - 7442	+	96	Hypothetical protein	*Vibrio* phage phi-pp2 (2e-65)	100	0	N
	21	7444 - 7725	+	93	Hypothetical protein	*Vibrio* phage V09 (2e-63)	100	0	N
	22	7722 - 7814	+	30	Hypothetical protein	*Vibrio* phage phi-pp2 (6e-3)	100	0	N
Metabolism	23	8003 - 8419	+	138	Endonuclease V [IPR004260; Pyr-dimer_DNA_glycosylase; Pyrimidine dimer DNA glycosylase]	*Vibrio* phage phi-pp2 (1e-100)	100	0	N
	24	8430 - 8861	+	143	Hypothetical protein	*Vibrio* phage phi-pp2 (2e-102)	100	1	N
	25	8858 - 9211	+	117	Hypothetical protein	*Vibrio* phage KVP40 (9e-80)	100	0	Y
	26	9465 - 9653	+	62	Hypothetical protein	*Vibrio* phage KVP40 (1e-38)	100	0	N
	27	9861 - 10085	+	74	Hypothetical protein	*Vibrio* phage phi-pp2 (2e-45)	100	0	N
	28	10082 - 10465	+	127	Hypothetical protein	*Vibrio* phage phi-pp2 (3e-90)	100	0	N
Metabolism	29	10450 - 11424	+	324	Trifunctional NAD biosynthesis/regulator protein NadR [IPR038727; NadR/Ttd14_AAA_dom; NadR/Ttd14, AAA domain]	*Vibrio* phage PVA8 (0.0)	96.6	0	N
	30	11418 - 11762	+	114	Hypothetical protein	*Vibrio* phage V09 (e-72)	97.4	3	N
	31	11759 - 12025	+	88	Hypothetical protein	*Vibrio* phage V05 (3e-54)	100	0	N

Metabolism	32	12050 - 12715	+	221	PnuC-like nicotinamide mononucleotide transport [IPR006419; NMN_transpt_PnuC; Nicotinamide mononucleotide transporter PnuC]	*Vibrio* phage KVP40 (9e-160)	100	7	N
	33	12712 - 12960	+	82	Hypothetical protein	*Vibrio* phage KVP40 (1e-53)	100	0	N
	34	12950 - 13237	+	95	Hypothetical protein	*Vibrio* phage V09 (2e-61)	98.9	0	N
	35	13234 - 13731	+	165	Hypothetical protein	*Vibrio* phage V09 (4e-118)	100	0	N
	36	13771 - 13965	+	64	Hypothetical protein	*Vibrio* phage Va3 (3e-37)	100	2	N
	37	13967 - 14236	+	89	Hypothetical protein	*Vibrio* phage V09 (4e-60)	100	0	N
	38	14236 - 14454	+	72	Hypothetical protein	*Vibrio* phage V09 (3e-42)	100	2	N
	39	14569 - 14931	+	120	Hypothetical protein	*Vibrio* phage KVP40 (7e-84)	100	0	N
	40	14933 - 15163	+	76	Hypothetical protein	*Vibrio* phage KVP40 (5e-48)	100	0	N
	41	15175 - 15414	+	79	Hypothetical protein	*Vibrio* phage KVP40 (2e-46)	100	0	N
	42	15416 - 15682	+	88	Hypothetical protein	*Vibrio* phage KVP40 (4e-56)	100	2	N
	43	15679 - 15846	+	55	Hypothetical protein	*Vibrio* phage KVP40 (1e-29)	100	1	Y
	44	15833 - 16570	+	245	Hypothetical protein	*Vibrio* phage KVP40 (0.0)	99.6	0	Y
	45	16645 - 16962	+	105	Hypothetical protein	*Vibrio* phage KVP40 (4e-71)	100	0	N
	46	16962 - 17336	+	124	Hypothetical protein	*Vibrio* phage KVP40 (4e-85)	99.2	0	N
	47	17339 - 17701	+	120	Hypothetical protein	*Vibrio* phage KVP40 (1e-84)	100	0	N
	48	17702 - 17881	+	59	Hypothetical protein	*Vibrio* phage KVP40 (6e-35)	100	0	N
	49	17943 - 18233	+	96	Hypothetical protein	*Vibrio* phage KVP40 (2e-61)	100	0	N
	50	18244 - 18696	+	150	Hypothetical protein	*Vibrio* phage KVP40 (4e-107)	100	0	N
	51	18699 - 18965	+	88	Hypothetical protein	*Vibrio* phage KVP40 (2e-58)	100	1	N
	52	18965 - 19243	+	92	Hypothetical protein	*Vibrio* phage KVP40 (2e-59)	100	0	N
	53	19245 - 20069	+	274	Hypothetical protein	*Vibrio* phage KVP40 (0.0)	100	0	N
	54	20069 - 20551	+	160	Hypothetical protein	*Vibrio* phage KVP40 (8e-116)	100	0	N
Metabolism	55	20554 - 21186	+	210	DNA endonuclease	*Vibrio* phage KVP40 (6e-155)	100	0	N
	56	21183 - 21797	+	204	Hypothetical protein	*Vibrio* phage KVP40 (1e-149)	100	0	N
	57	21805 - 22332	+	175	Hypothetical protein	*Vibrio* phage V09 (2e-128)	100	0	N
	58	22329 - 22577	+	82	Hypothetical protein	*Vibrio* phage V09 (5e-53)	98.8	0	N
	59	22561 - 22911	+	116	Hypothetical protein	*Vibrio* phage V09 (7e-79)	100	0	N
	60	23033 - 23362	+	109	Hypothetical protein	*Vibrio* phage V09 (3e-75)	100	0	N
	61	23366 - 23713	+	115	Hypothetical protein	*Vibrio* phage KVP40 (3e-78)	100	0	N
	62	23706 - 24140	+	144	Hypothetical protein	*Vibrio* phage KVP40 (4e-101)	100	0	N
	63	24137 - 24601	+	154	Hypothetical protein	*Vibrio* phage V09 (2e-112)	100	0	N
	64	24598 - 25068	+	156	Hypothetical protein	*Vibrio* phage KVP40 (1e-110)	99.4	1	N
	65	25065 - 25394	+	109	Hypothetical protein	*Vibrio* phage KVP40 (1e-73)	99.1	4	N
	66	25478 - 25726	+	82	Hypothetical protein	*Vibrio* phage KVP40 (7e-52)	97.6	0	N
	67	25736 - 26155	+	139	Hypothetical protein	*Vibrio* phage KVP40 (1e-96)	97.8	0	N
	68	26236 - 26931	+	231	Hypothetical protein	*Vibrio* phage KVP40 (6e-169)	99.6	0	Y
	69	26939 - 27721	+	260	Hypothetical protein	*Vibrio* phage V09 (0.0)	99.2	0	N
	70	27791 - 28321	+	176	Hypothetical protein	*Vibrio* phage V09 (3e-106)	100	0	N
	71	28334 - 28552	+	82	Hypothetical protein	*Vibrio* phage KVP40 (6e-41)	100	0	N
	72	28536 - 29390	+	284	Hypothetical protein	*Vibrio* phage V09 (0.0)	100	0	N

	73	29383 - 30015	+	210	Hypothetical protein	*Vibrio* phage KVP40 (4e-155)	99.5	0	N
Metabolism	74	30005 - 30688	+	227	QueC-like queuosine biosynthesis [IPR018317; QueC; Queuosine biosynthesis protein QueC]	*Vibrio* phage KVP40 (0.0)	100	0	N
	75	30663 - 31556	+	297	Hypothetical protein [IPR016471; Nicotinamide_PRibTrfase; Nicotinamide phosphoribosyl transferase]	*Vibrio* phage KVP40 (0.0)	100	0	N
	76	31559 - 32548	+	329	Hypothetical protein [IPR007197; rSAM; Radical SAM]	*Vibrio* phage KVP40 (0.0)	100	0	N
	77	32541 - 33665	-	374	Hypothetical protein [IPR007197; rSAM; Radical SAM]	*Vibrio* phage KVP40 (0.0)	100	0	N
	78	33676 - 34248	-	190	Hypothetical protein	*Vibrio* phage KVP40 (2e-136)	98.6	0	N
	79	34383 - 34619	+	78	Hypothetical protein	*Vibrio* phage KVP40 (3e-47)	100	0	Y
Metabolism	80	34719 - 36212	+	497	Nicotinamide phosphoribosyl transferase [IPR016471; Nicotinamide_PRibTrfase; Nicotinamide phosphoribosyl transferase]	*Vibrio* phage KVP40 (3e-47)	100	0	N
	81	36320 - 36646	+	108	Hypothetical protein	*Vibrio phage KVP40 (3e-73)*	100	0	N
	82	36688 - 36930	+	80	Hypothetical protein	*Vibrio* phage KVP40 (9e-51)	100	0	N
	83	36937 - 37176	+	79	Hypothetical protein	*Vibrio* phage KVP40 (2e-52)	100	0	N
	84	37176 - 38444	+	422	Hypothetical protein	*Vibrio* phage KVP40 (0.0)	100	0	N
	85	38505 - 38765	+	86	Hypothetical protein	*Vibrio* phage KVP40 (5e-58)	100	0	N
	86	38777 - 39496	+	239	Hypothetical protein	*Vibrio* phage phi-pp2 (5e-174)	100	0	Y
	87	39615 - 39923	+	102	Hypothetical protein	*Vibrio* phage KVP40 (5e-70)	100	0	N
	88	39949 - 41001	-	350	Hypothetical protein [IPR007197; rSAM; Radical SAM]	*Vibrio* phage KVP40 (0.0)	99.7	0	N
	89	41001 - 41273	-	90	Hypothetical protein	*Vibrio* phage KVP40 (1e-57)	100	0	N
	90	41270 - 42271	-	333	Hypothetical protein	*Vibrio* phage phi-pp2 (0.0)	100	0	N
	91	42341 - 42547	+	68	Hypothetical protein	*Vibrio* phage KVP40 (9e-41)	100	0	N
	92	42549 - 43025	+	158	Hypothetical protein	*Vibrio* phage KVP40 (1e-110)	99.4	0	N
	93	43058 - 45283	+	741	Ribonucleoside diphosphate reductase large subunit [IPR039718; Rrm1; Ribonucleoside-diphosphate reductase large subunit]	*Vibrio* phage KVP40 (0.0)	99.9	0	N
Metabolism	94	45293 - 46417	+	374	Ribonucleoside diphosphate reductase small subunit [IPR000358; RNR_small_fam; Ribonucleotide reductase small subunit family]	*Vibrio* phage KVP40 (0.0)	100	0	N
	95	46419 - 46718	+	99	Thioredoxin [IPR002109; Glutaredoxin; Glutaredoxin]	*Vibrio* phage KVP40 (2e-138)	100	0	N
Lysis	96	46793 - 47365	+	190	Transglycosylase SLT domain protein [IPR008258; Transglycosylase_SLT_dom_1; Transglycosylase SLT domain 1]	*Vibrio* phage KVP40 (2e-138)	100	0	Y
	97	47365 - 47550	+	61	Hypothetical protein	*Vibrio* phage KVP40 (5e-37)	100	0	N
Metabolism	98	47651 - 48655	+	334	Thioredoxin [IPR003593; AAA+_ATPase; AAA+ ATPase domain]	*Vibrio* phage V09 (0.0)	100	0	N
	99	48701 - 49987	+	428	DNA topoisomerase II medium subunit [IPR002205; Topo_IIA_dom_A; DNA topoisomerase, type IIA, domain A]	*Vibrio* phage KVP40 (0.0)	99.8	0	N
	100	49987 - 50229	+	80	Hypothetical protein	*Vibrio* phage KVP40 (2e-52)	100	0	N
Metabolism	101	50238 - 51119	+	293	QueE-like radical SAM domain [IPR024924; 7-CO-7-deazaguanine_synth-like; 7-carboxy-7-deazaguanine synthase-like]	*Vibrio* phage V09 (0.0)	100	0	N
	102	51155 - 51409	+	84	Hypothetical protein	*Vibrio* phage KVP40 (7e-53)	100	3	N
	103	51475 - 51633	+	52	Hypothetical protein	*Vibrio* phage KVP40 (9e-28)	100	0	N
	104	51623 - 51922	+	99	Hypothetical protein	*Vibrio* phage KVP40 (1e-68)	100	0	N
	105	51915 - 52184	+	89	Hypothetical protein	*Vibrio* phage KVP40 (4e-58)	100	0	N
	106	52192 - 52464	+	90	Hypothetical protein	*Vibrio* phage KVP40 (5e-54)	100	0	N
	107	52579 - 53697	+	372	Hypothetical protein	*Vibrio* phage KVP40 (0.0)	100	0	N
	108	53739 - 54224	+	161	Hypothetical protein	*Vibrio* phage KVP40 (2e-118)	100	0	N
	109	54289 - 54996	+	234	Hypothetical protein [IPR003714; PhoH; PhoH-like protein]	*Vibrio* phage KVP40 (9e-174)	100	0	N

	110	54996 - 55304	+	102	Hypothetical protein	*Vibrio* phage V09 (6e-68)	100	0	N
	111	55539 - 56270	+	243	Hypothetical protein	*Vibrio* phage KVP40 (0.0)	100	0	N
Metabolism	112	56274 - 56573	+	99	Anti-sigma factor [IPR015100; AsiA; Anti-Sigma Factor A]	*Vibrio* phage KVP40 (0.0)	100	0	N
	113	56582 - 56878	+	98	Hypothetical protein	*Vibrio* phage V09 (3e-67)	100	0	N
	114	56919 - 57494	-	191	Hypothetical protein	*Vibrio* phage PVA8 (3e-133)	100	1	N
Structure	115	57545 - 60802	-	1085	Long tail fiber protein distal subunit	*Vibrio* phage V09 (0.0)	99.7	0	N
	116	60874 - 64149	-	1091	Long tail fiber protein distal subunit [IPR005601; Tail_fibre_p36; Tail fibre protein p36]	*Vibrio* phage V09 (0.0)	100	0	N
	117	64214 - 64738	-	174	Hypothetical protein	*Vibrio* phage KVP40 (2e-126)	100	0	N
	118	64789 - 66225	-	478	Hypothetical protein	*Vibrio* phage V09 (0.0)	100	0	N
	119	66317 - 66631	-	104	Hypothetical protein	*Vibrio* phage V09 (3e-70)	100	0	N
	120	66628 - 66963	-	111	Hypothetical protein	*Vibrio* phage KVP40 (7e-77)	100	0	N
	121	66956 - 67330	-	124	Hypothetical protein	*Vibrio* phage V09 (3e-87)	100	0	N
	122	67312 - 67668	-	118	Hypothetical protein [IPR046909; cREC_REC; Cyclic-phosphate processing, Receiver domain]	*Vibrio* phage KVP40 (6e-80)	100	0	N
	123	67731 - 68102	+	123	Hypothetical protein	*Vibrio* phage V09 (1e-82)	100	0	N
	124	68134 - 68346	-	70	Hypothetical protein	*Vibrio* phage KVP40 (2e-42)	100	0	N
	125	68445 - 68675	-	76	Hypothetical protein	*Vibrio* phage KVP40 (4e-46)	100	0	N
	126	68675 - 68881	-	68	Hypothetical protein	*Vibrio* phage KVP40 (1e-36)	100	0	N
	127	68919 - 69296	-	125	Hypothetical protein	*Vibrio* phage KVP40 (1e-85)	100	0	N
	128	69736 - 69915	-	59	Hypothetical protein	*Vibrio* phage KVP40 (4e-33)	100	0	N
	129	70751 - 70990	-	79	Hypothetical protein	*Vibrio* phage KVP40 (2e-51)	100	0	N
	130	71171 - 71365	-	64	Hypothetical protein	*Vibrio* phage KVP40 (1e-38)	100	0	N
	131	72170 - 72379	-	69	Hypothetical protein	*Vibrio* phage V09 (7e-41)	100	0	N
	132	75019 - 75132	-	37	Hypothetical protein	*Vibrio* phage phi-pp2 (3e-19)	100	0	N
	133	75259 - 75492	-	77	Hypothetical protein	*Vibrio* phage KVP40 (1e-47)	100	0	N
	134	75639 - 76160	-	173	Hypothetical protein	*Vibrio* phage phi-pp2 (9e-123)	99.4	0	N
	135	76421 - 76651	-	76	Hypothetical protein	*Vibrio* phage phi-pp2 (9e-46)	98.7	0	N
	136	76809 - 77177	-	122	Hypothetical protein	*Vibrio* phage phi-pp2 (4e-85)	100	0	N
	137	78382 - 78636	-	84	Hypothetical protein	*Vibrio* phage phi-pp2 (2e-51)	100	0	N
Metabolism	138	78629 - 79084	-	151	RNA ligase	*Vibrio* phage KVP40 (5e-107)	100	0	N
Structure	139	79084 - 79350	-	88	Tail fiber chaperone	*Vibrio* phage KVP40 (7e-55)	100	0	N
Metabolism	140	79360 - 79998	-	212	Deoxynucleoside monophosphate kinase [IPR048444; DNMK; Deoxynucleotide monophosphate kinase]	*Vibrio* phage KVP40 (3e-154)	100	0	N
Structure	141	80231 - 80764	-	177	Tail completion and sheath stabilizer protein [IPR010667; Phage_T4_Gp19; Bacteriophage T4, Gp19, tail tube]	*Vibrio* phage KVP40 (2e-129)	100	0	N
	142	80764 - 82734	-	656	Hypothetical protein	*Vibrio* phage phi-pp2 (0.0)	100	0	N
	143	82734 - 82901	-	55	Hypothetical protein	*Vibrio* phage KVP40 (2e-31)	100	0	N
Structure	144	82901 - 83749	-	282	Baseplate hub assembly catalyst [IPR024364; Baseplate_phage_T4-like; Baseplate hub assembly protein, bacteriophage T4-like]	*Vibrio* phage KVP40 (0.0)	100	0	N
	145	83761 - 84507	-	248	Baseplate-tail tube initiator [IPR010667;Phage_T4_Gp19; Bacteriophage T4, Gp19, tail tube]	*Vibrio* phage KVP40 (0.0)	100	0	N
Packaging	146	84511 - 85107	-	198	DNA end protector	*Vibrio* phage VH1_2019 (8e-146)	100	0	N

	147	85107 - 85562	-	151	Head completion nuclease [IPR046390; NUCL_HEAD_T4; Head completion nuclease]	*Vibrio* phage KVP40 (5e-107)	100	0	N
Structure	148	85629 - 86768	+	379	Baseplate tail tube cap [IPR024389; Gp48_T4-like; Baseplate tail-tube protein gp48, T4-like virus]	*Vibrio* phage KVP40 (0.0)	99.7	0	N
	149	86768 - 87346	+	192	Baseplate wedge subunit [IPR022607; Phage_T4_Gp53_baseplate_wedge; Baseplate wedge protein gp53, bacteriophage T4]	*Vibrio* phage KVP40 (1e-138)	100	0	N
	150	87348 - 88619	+	423	Baseplate hub protein	*Vibrio* phage KVP40 (0.0)	100	0	N
	151	88619 - 89884	+	421	Baseplate hub subunit and tail lysozyme [IPR009590; Gp5_OB_N; Protein Gp5, N-terminal OB-fold domain]	*Vibrio* phage V09 (0.0)	99.8	0	N
	152	89889 - 90374	+	161	Hypothetical protein	*Vibrio* phage KVP40 (4e-115)	100	0	N
	153	90374 - 90670	+	98	Hypothetical protein	*Vibrio* phage KVP40 (3e-63)	100	0	Y
	154	90660 - 91019	-	119	Hypothetical protein	*Vibrio* phage KVP40 (5e-77)	100	2	N
	155	91074 - 91949	-	291	Hypothetical protein	*Vibrio* phage KVP40 (0.0)	100	0	Y
Structure	156	92135 - 92554	+	139	Baseplate wedge subunit [IPR007048; IraD/Gp25-like; IraD/Gp25-like]	*Vibrio* phage KVP40 (1e-96)	100	0	N
	157	92639 - 94597	+	652	Baseplate wedge subunit [IPR034698; GP6_T4; Baseplate wedge protein gp6]	*Vibrio* phage KVP40 (0.0)	100	0	N
	158	94597 - 98094	+	1165	Baseplate wedge initiator [IPR048810; Gp7_helical; Baseplate wedge protein gp7, helical domain]	*Vibrio* phage PVA8 (0.0)	99.9	0	N
	159	98096 - 99118	+	340	Baseplate wedge subunit [IPR015298; Phage_T4_Gp8; Bacteriophage T4, Gp8]	*Vibrio* phage PVA8 (0.0)	100	0	N
	160	99174 - 100130	+	318	Baseplate wedge tail fiber connector [IPR008987; Baseplate_struct_prot_Gp9/10; Baseplate structural protein Gp9/Gp10]	*Vibrio* phage KVP40 (0.0)	99.7	0	N
	161	100140 - 102386	+	748	Baseplate wedge subunit [IPR008987; Baseplate_struct_prot_Gp9/10; Baseplate structural protein Gp9/Gp10]	*Vibrio* phage KVP40 (0.0)	100	0	N
	162	102386 - 103072	+	228	Baseplate wedge subunit [IPR014791; Baseplate_struct_Gp11; Baseplate structural protein Gp11]	*Vibrio* phage KVP40 (8e-167)	100	0	N
	163	103072 - 104610	+	512	Tail collar fiber protein	*Vibrio* phage KVP40 (0.0)	100	0	N
	164	104607 - 106028	+	473	Tail collar fiber protein	*Vibrio* phage KVP40 (0.0)	99.6	0	N
	165	106060 - 106254	-	64	Hypothetical protein	*Vibrio* phage PG216 (5e-37)	98.4	0	N
Structure	166	106327 - 108006	+	559	Fibritin neck whisker protein	*Vibrio* phage V09 (0.0)	99.8	0	N
	167	108017 - 108940	+	307	Neck protein	*Vibrio* phage V09 (0.0)	100	0	N
	168	108944 - 109780	+	278	Head closure Hc2 [IPR021674; Phage_T4_Gp14_neck-protein; Bacteriophage T4, Gp14, neck protein]	*Vibrio* phage KVP40 (0.0)	100	0	N
	169	109838 - 111142	+	434	Tail sheath stabilizer and completion protein [IPR031997; T4-gp15_tss; Myoviridae tail sheath stabiliser]	*Vibrio* phage V09 (0.0)	99.3	0	N
	170	111197 - 111745	+	182	Hypothetical protein	*Vibrio* phage KVP40 (5e-126)	100	0	N
Packaging	171	111857 - 112405	+	182	Terminase small subunit [IPR020342; Phage_T4_Gp16_DNA-pack; Bacteriophage T4, Gp16, DNA-packaging]	*Vibrio* phage KVP40 (3e-125)	100	0	N
	172	112365 - 114167	+	600	Terminase large subunit [IPR044267; Terminase_large_su_gp17-like; Terminase, large subunit, gp17-like]	*Vibrio* phage KVP40 (0.0)	100	0	N
Structure	173	114214 - 116229	+	671	Tail sheath protein	*Vibrio* phage KVP40 (0.0)	99.7	0	N
	174	116280 - 116780	+	166	Tail tube protein [IPR010667; Phage_T4_Gp19; Bacteriophage T4, Gp19, tail tube]	*Vibrio* phage KVP40 (7e-120)	100	0	N
Packaging	175	116820 - 118367	+	515	Portal protein [IPR010823; Portal_Gp20; Portal protein Gp20]	*Vibrio* phage V09 (0.0)	100	0	N
Structure	176	118380 - 118547	+	55	Prohead core protein	*Vibrio* phage KVP40 (1e-29)	100	0	N

	177	118547 - 119038	+	163	Head scaffolding protein [IPR016415; Phage_T4_Gp68; Bacteriophage T4, Prohead assembly protein gp68]	*Vibrio* phage V09 (6e-112)	100	0	N
Packaging	178	119041 - 119682	+	213	Head maturation protease [IPR005082; Peptidase_U9_T4_prohead; Peptidase U9, T4 prohead protease]	*Vibrio* phage KVP40 (5e-152)	100	0	N
Structure	179	119715 - 120557	+	280	Head scaffolding protein	*Vibrio* phage KVP40 (0.0)	100	0	N
	180	120628 - 122172	+	514	Major capsid protein [IPR038997; CAPSID_Myoviridae; Major capsid protein, Myoviridae]	*Vibrio* phage VH1_2019 (0.0)	100	1	N
Metabolism	181	122229 - 123326	-	365	tRNA nucleotidyltransferase [IPR002646; PolA_pol_head_dom; Poly A polymerase, head domain]	*Vibrio* phage phi-pp2 (0.0)	99.7	0	N
	182	123398 - 123739	+	113	Hypothetical protein	*Vibrio* phage phi-pp2 (4e-78)	100	0	N
	183	123749 - 125203	+	484	Hypothetical protein	*Vibrio* phage PVA8 (0.0)	98.4	0	N
Packaging	184	125240 - 125731	+	163	Prohead protease inhibitor	*Vibrio* phage V09 (2e-112)	100	0	N
	185	125740 - 126072	+	110	Hypothetical protein	*Vibrio* phage VH1_2019 (3e-73)	100	0	N
	186	126131 - 127315	-	394	Hypothetical protein	*Vibrio* phage V09 (0.0)	100	0	N
	187	127426 - 128388	-	320	Hypothetical protein	*Vibrio* phage V09 (0.0)	99.7	0	N
	188	128391 - 128984	-	197	Hypothetical protein	*Vibrio* phage KVP40 (2e-141)	99.5	0	N
	189	129067 - 129693	-	208	Hypothetical protein	*Vibrio* phage KVP40 (6e-147)	100	0	N
	190	129710 - 129985	-	91	Hypothetical protein	*Vibrio* phage V09 (1e-59)	100	0	N
	191	130047 - 130535	-	162	Hypothetical protein	*Vibrio* phage KVP40 (4e-114)	100	0	N
Packaging	192	130540 - 131049	-	169	Prohead protease inhibitor	*Vibrio* phage KVP40 (5e-117)	98.3	0	N
	193	131058 - 131264	-	68	Hypothetical protein	*Vibrio* phage KVP40 (1e-42)	100	0	N
Metabolism	194	131328 - 132851	+	507	ATP-dependent DNA helicase [IPR001650; Helicase_C; Helicase, C-terminal]	*Vibrio* phage V09 (0.0)	99.6	0	N
	195	133004 - 133174	-	56	Hypothetical protein	*Vibrio* phage KVP40 (1e-31)	100	0	N
	196	133174 - 133347	-	57	Hypothetical protein	*Vibrio* phage KVP40 (3e-33)	100	0	N
Metabolism	197	133349 - 133762	-	137	tRNA amidotransferase [IPR003789; Asn/Gln_tRNA_amidoTrase-B-like; Aspartyl/glutamyl-tRNA amidotransferase subunit B-like]	*Vibrio* phage phi-pp2 (1e-92)	100	0	N
	198	133759 - 134172	-	137	UvsY-like recombination mediator [IPR021289; UvsY; Recombination, repair and ssDNA binding protein UvsY]	*Vibrio* phage KVP40 (5e-94)	100	0	N
Structure	199	134340 - 137024	-	894	Hinge connector of long tail fiber protein distal connector	*Vibrio* phage PG216 (0.0)	99.8	0	N
	200	137033 - 140803	-	1256	Long tail fiber proximal subunit	*Vibrio* phage V09 (0.0)	99.9	0	N
Metabolism	201	140883 - 141815	+	310	RNase H [IPR038969; FEN; Flap endonuclease]	*Vibrio* phage KVP40 (0.0)	100	0	N
	202	141898 - 142170	+	90	Transcriptional regulator [PF11126; Phage_DsbA; Transcriptional regulator DsbA]	*Vibrio* phage KVP40 (1e-56)	100	0	N
	203	142160 - 142453	+	97	Late promoter transcription coactivator [IPR031836; Trans_coact; Late transcription coactivator]	*Vibrio* phage KVP40 (1e-64)	100	0	N
	204	142419 - 143045	+	208	DNA helicase loader [IPR008944; Phage_T4_Gp59; Bacteriophage T4, Gp59, helicase assembly protein]	*Vibrio* phage phi-pp2 (9e-152)	99.5	0	N
	205	143096 - 144010	+	304	Single strand DNA binding protein [IPR046395; SSB_T4; Bacteriophage T4, Gp32, single-stranded DNA-binding]	*Vibrio* phage V09 (0.0)	99.7	0	N
	206	144061 - 144606	+	181	Dihydrofolate reductase	*Vibrio* phage KVP40 (2e-132)	100	0	N
	207	144603 - 145322	+	239	ATP-dependent protease [IPR023562; ClpP/TepA; Clp protease proteolytic subunit /Translocation-enhancing protein TepA]	*Vibrio* phage PG216 (1e-177)	100	0	N
	208	145389 - 146489	+	366	DNA repair protein [IPR013765; DNA_recomb/repair_RecA; DNA recombination and repair protein RecA]	*Vibrio* phage KVP40 (0.0)	99.7	0	N
Structure	209	146549 - 146851	+	100	Head vertex assembly chaperone [IPR021049; Phage_T4_Gp40; Bacteriophage T4, Gp40, head assembly]	*Vibrio* phage KVP40 (4e-65)	100	0	N

Metabolism	210	146905 - 148188	+	427	DNA primase-helicase [IPR046393; Helic_T4; Bacteriophage T4 DnaB-like replicative helicase]	*Vibrio* phage KVP40 (0.0)	100	0	N
	211	148182 - 148427	+	81	Hypothetical protein	*Vibrio* phage phi-pp2 (2e-51)	100	0	N
Metabolism	212	148428 - 150263	+	611	Anaerobic ribonucleoside reductase large subunit [IPR012833; NrdD; Ribonucleoside-triphosphate reductase, anaerobic]	*Vibrio* phage KVP40 (0.0)	99.8	0	N
	213	150381 - 150800	+	139	Hypothetical protein	*Vibrio* phage PVA8 (5e-99)	100	0	N
	214	150800 - 151369	+	189	Hypothetical protein	*Vibrio* phage KVP40 (9e-137)	100	0	N
Metabolism	215	151350 - 151826	+	158	Anaerobic ribonucleotide reductase small subunit [IPR012837; NrdG; Ribonucleoside-triphosphate reductase activating, anaerobic]	*Vibrio* phage KVP40 (1e-114)	100	0	N
	216	151826 - 152350	+	174	Hypothetical protein	*Vibrio* phage phi-pp2 (2e-126)	99.4	0	N
	217	152415 - 153260	+	281	Hypothetical protein	*Vibrio* phage VH1_2019 (0.0)	100	0	N
Metabolism	218	153264 - 154088	+	274	DNA helicase	*Vibrio* phage V09 (0.0)	100	0	N
	219	154088 - 154564	+	158	Hypothetical protein	*Vibrio* phage V09 (0.0)	100	0	N
Metabolism	220	154645 - 155703	+	352	DNA primase [IPR046392; PRIMASE_T4; DNA primase, bacteriophage T4]	*Vibrio* phage KVP40 (0.0)	100	0	N
	221	155703 - 156200	+	165	dUTPase [IPR008181; dUTPase; Deoxyuridine triphosphate nucleotidohydrolase]	*Vibrio* phage phi-pp2 (3e-114)	99.4	0	N
	222	156200 - 156436	+	78	Hypothetical protein	*Vibrio* phage KVP40 (2e-46)	100	0	N
Metabolism	223	156433 - 157125	+	230	exonuclease A [IPR033390; Rv2179c-like; 3'-5' exoribonuclease Rv2179c-like domain]	*Vibrio* phage KVP40 (3e-171)	100	0	N
	224	157122 - 157283	+	53	Hypothetical protein	*Vibrio* phage KVP40 (1e-30)	100	0	N
	225	157352 - 157489	+	45	Hypothetical protein	*Vibrio* phage KVP40 (2e-23)	100	0	N
	226	157486 - 157884	+	132	Hypothetical protein	*Vibrio* phage KVP40 (3e-93)	100	0	N
	227	157871 - 158116	+	81	Hypothetical protein	*Vibrio* phage KVP40 (5e-52)	100	0	N
	228	158113 - 158556	+	147	Hypothetical protein	*Vibrio* phage KVP40 (7e-106)	100	0	N
	229	158549 - 158779	+	76	Hypothetical protein	*Vibrio* phage KVP40 (1e-47)	100	0	N
	230	158816 - 159016	+	66	Hypothetical protein	*Vibrio* phage KVP40 (2e-38)	100	1	N
Metabolism	231	159026 - 159925	+	299	Thymidylate synthase [IPR045097; Thymidate_synth/dCMP_Mease; Thymidylate synthase/dCMP hydroxymethylase]	*Vibrio* phage KVP40 (0.0)	100	0	N
	232	159922 - 160116	+	64	Hypothetical protein	*Vibrio* phage KVP40 (4e-37)	100	0	N
	233	160119 - 160325	+	68	Hypothetical protein	*Vibrio* phage KVP40 (2e-36)	100	2	N
	234	160325 - 160615	+	96	Hypothetical protein	*Vibrio* phage KVP40 (2e-64)	100	0	N
	235	160612 - 160932	+	106	Hypothetical protein	*Vibrio* phage KVP40 (3e-69)	100	0	N
	236	160933 - 161229	+	98	Hypothetical protein	*Vibrio* phage phi-pp2 (2e-62)	100	0	N
	237	161229 - 161990	+	253	Hypothetical protein	*Vibrio* phage KVP40 (0.0)	99.6	0	N
	238	162060 - 162389	+	109	Hypothetical protein	*Vibrio* phage KVP40 (1e-74)	100	0	N
	239	162392 - 162793	+	133	Hypothetical protein	*Vibrio* phage KVP40 (5e-92)	100	0	N
Metabolism	240	162797 - 163504	+	235	HNH endonuclease	*Vibrio* phage KVP40 (9e-175)	100	0	N
	241	163506 - 163766	+	86	Hypothetical protein	*Vibrio* phage KVP40 (2e-56)	100	0	N
	242	163763 - 164131	+	122	Hypothetical protein	*Vibrio* phage KVP40 (4e-86)	100	0	N
Metabolism	243	164128 - 164868	+	246	Sir2 (NAD-dependent deacetylase) [IPR003000; Sirtuin; Sirtuin family]	*Vibrio* phage KVP40 (0.0)	100	0	N
	244	165024 - 166817	+	579	DNA topoisomerase II large subunit [IPR001241; Topo_IIA; DNA topoisomerase, type IIA]	*Vibrio* phage KVP40 (0.0)	100	0	N
	245	166814 - 167044	+	76	Hypothetical protein	*Vibrio* phage KVP40 (3e-46)	100	0	N
	246	167044 - 167259	+	71	Hypothetical protein	*Vibrio* phage KVP40 (1e-42)	100	0	N

	247	167277 - 167543	+	88	Hypothetical protein	*Vibrio* phage KVP40 (2e-54)	100	0	N
	248	167554 - 167946	+	130	Hypothetical protein	*Vibrio* phage PVA8 (2e-89)	97.7	0	N
	249	167943 - 168206	+	87	Hypothetical protein	*Vibrio* phage phi-pp2 (1e-55)	100	0	N
	250	168194 - 168922	+	242	Hypothetical protein [IPR004843; Calcineurin-like_PHP_ApaH; Calcineurin-like phosphoesterase domain, ApaH type]	*Vibrio* phage V09 (0.0)	100	0	N
	251	168919 - 169107	+	62	Hypothetical protein	*Vibrio* phage PVA8 (2e-32)	98.4	1	Y
	252	169153 - 169398	+	81	Hypothetical protein	*Vibrio* phage KVP40 (2e-50)	100	0	N
	253	169459 - 169971	+	170	Hypothetical protein	*Vibrio* phage V09 (0.0)	100	0	N
	254	170041 - 170637	+	198	Hypothetical protein	*Vibrio* phage KVP40 (2e-144)	100	0	N
	255	170682 - 170993	+	103	Hypothetical protein	*Vibrio* phage KVP40 (3e-71)	100	0	N
Metabolism	256	171125 - 172468	+	447	DNA ligase [IPR012310; DNA_ligase_ATP-dep_cent; DNA ligase, ATP-dependent, central]	*Vibrio* phage PVA8 (0.0)	100	0	N
	257	172525 - 173406	+	293	Hypothetical protein	*Vibrio* phage VH1_2019 (0.0)	100	0	N
Metabolism	258	173437 - 175101	-	554	RNA polymerase-ADP-ribosyltransferase	*Vibrio* phage PVA8 (0.0)	100	0	N
	259	175140 - 175766	+	208	Hypothetical protein [IPR004843; Calcineurin-like_PHP_ApaH; Calcineurin-like phosphoesterase domain, ApaH type]	*Vibrio* phage V09 (1e-152)	100	0	N
	260	175875 - 176693	+	272	Hypothetical protein [IPR006640; SprT-like_domain; SprT-like]	*Vibrio* phage PVA8 (0.0)	100	0	N
Metabolism	261	176851 - 177090	+	79	Glutaredoxin [IPR036249; Thioredoxin-like_sf; Thioredoxin-like superfamily]	*Vibrio* phage phi-pp2 (3e-53)	100	0	N
Structure	262	177149 - 178045	+	298	Capsid vertex protein [IPR010762; Gp23/Gp24_T4-like; Capsid protein, T4-like bacteriophage-like]	*Vibrio* phage V09 (0.0)	100	0	N
Metabolism	263	178054 - 178566	+	170	RNA polymerase sigma factor for late transcription [IPR046386; T4_sigma-like_factor; RNA polymerase sigma-like factor]	*Vibrio* phage KVP40 (1e-125)	100	0	N
	264	178576 - 180021	+	481	Hypothetical protein	*Vibrio* phage PVA8 (0.0)	99.8	0	N
	265	180030 - 180677	+	215	Hypothetical protein	*Vibrio* phage V09 (6e-157)	100	0	N
	266	180769 - 181026	+	85	Hypothetical protein	*Vibrio* phage KVP40 (1e-55)	100	0	N
	267	181074 - 181532	+	152	Hypothetical protein	*Vibrio* phage KVP40 (5e-108)	100	0	N
	268	181532 - 181882	+	116	Hypothetical protein	*Vibrio* phage KVP40 (6e-78)	100	0	N
	269	181882 - 182346	+	154	Hypothetical protein [IPR012596; Phage_T4_Y12G; Bacteriophage T4, Y12G]	*Vibrio* phage phi-pp2 (9e-112)	99.4	0	N
Metabolism	270	182324 - 182836	+	170	5'(3')-deoxyribonucleotidase [IPR010708; 5'(3')-deoxyribonucleotidase; 5'(3')-deoxyribonucleotidase]	*Vibrio* phage VH1_2019 (5e-126)	100	0	N
	271	182833 - 183873	+	346	SbcD-like subunit of palindrome specific endonuclease [IPR004843; Calcineurin-like_PHP_ApaH; Calcineurin-like phosphoesterase domain, ApaH type]	*Vibrio* phage KVP40 (0.0)	99.7	0	N
	272	183873 - 184097	+	74	Hypothetical protein	*Vibrio* phage KVP40 (6e-49)	100	0	N
Metabolism	273	184090 - 186327	+	745	SbcC-like subunit of palindrome specific endonuclease	*Vibrio* phage V09 (0.0)	99.9	0	N
	274	186324 - 186788	+	154	Hypothetical protein	*Vibrio* phage KVP40 (2e-113)	100	0	N
	275	186845 - 187054	+	69	Hypothetical protein	*Vibrio* phage KVP40 (4e-45)	100	0	N
Metabolism	276	187089 - 187754	+	221	Sliding clamp of DNA polymerase [IPR046389; Sliding_clamp_T4; Sliding clamp]	*Vibrio* phage V09 (2e-159)	100	0	N
	277	187869 - 188411	+	180	Hypothetical protein	*Vibrio* phage V09 (5e-134)	100	0	N
Metabolism	278	188401 - 189357	+	318	Clamp loader large subunit of DNA polymerase [IPR046388; T4_Clamp_Loader_L; Sliding-clamp-loader large subunit]	*Vibrio* phage V09 (0.0)	100	0	N
	279	189367 - 189855	+	162	Clamp loader small subunit of DNA polymerase [IPR031868; Phage_clamp_gp62; Sliding-clamp-loader small subunit gp62]	*Vibrio* phage KVP40 (7e-118)	100	0	N

	280	189890 - 190270	+	126	Translational repressor protein regA [IPR002702; Transl_repress_RegA; Translation repressor RegA]	*Vibrio* phage KVP40 (9e-89)	100	0	N
	281	190340 - 190858	+	172	Hypothetical protein	*Vibrio* phage KVP40 (3e-126)	100	0	N
Metabolism	282	190919 - 193471	+	850	DNA polymerase [IPR006172; DNA-dir_DNA_pol_B; DNA-directed DNA polymerase, family B]	*Vibrio* phage V09 (0.0)	100	0	N
	283	193464 - 193772	+	102	Hypothetical protein	*Vibrio* phage phi-pp2 (2e-70)	100	0	N
	284	193836 - 194981	+	381	Hypothetical protein	*Vibrio* phage KVP40 (0.0)	100	0	N
	285	194982 - 195254	+	90	Hypothetical protein	*Vibrio* phage KVP40 (3e-60)	100	0	N
Lysis	286	195291 - 195674	+	127	Rz-like spanin	*Vibrio* phage phi-pp2 (2e-87)	100	0	Y
	287	195664 - 195993	+	109	Rz-like spanin	*Vibrio* phage phi-pp2 (2e-74)	100	0	Y
	288	196073 - 196372	+	99	Hypothetical protein	*Vibrio* phage KVP40 (4e-66)	100	0	N
	289	196374 - 196607	+	77	Hypothetical protein	*Vibrio* phage KVP40 (2e-49)	100	0	N
Metabolism	290	196618 - 197535	+	305	3'-phosphatase 5'-polynucleotide kinase [IPR027417; P-loop_NTPase; P-loop containing nucleoside triphosphate hydrolase]	*Vibrio* phage phi-pp2 (0.0)	99.7	0	N
	291	197545 - 198090	+	181	Hypothetical protein	*Vibrio* phage KVP40 (1e-122)	100	1	Y
	292	198090 - 198602	+	170	Hypothetical protein [IPR002589; Macro_dom; Macro domain]	*Vibrio* phage KVP40 (1e-123)	100	0	N
	293	198773 - 199003	+	76	Hypothetical protein	*Vibrio* phage KVP40 (4e-49)	100	2	N
	294	198996 - 199187	+	63	Hypothetical protein	*Vibrio* phage KVP40 (1e-39)	100	0	N
	295	199180 - 199578	+	132	Hypothetical protein	*Vibrio* phage phi-pp2 (4e-90)	100	0	N
	296	199652 - 201106	+	484	Hypothetical protein	*Vibrio* phage VH1_2019 (0.0)	99.2	0	N
	297	201176 - 201439	+	87	Hypothetical protein	*Vibrio* phage KVP40 (4e-54)	100	0	N
	298	201450 - 202073	+	207	Hypothetical protein	*Vibrio* phage KVP40 (2e-152)	99.5	0	N
	299	202131 - 202346	+	71	Hypothetical protein	*Vibrio* phage KVP40 (6e-45)	100	0	N
	300	202346 - 202723	+	125	Hypothetical protein	*Vibrio* phage phi-pp2 (1e-90)	100	0	N
	301	202792 - 203412	+	206	Hypothetical protein	*Vibrio* phage V09 (1e-151)	99.5	0	N
	302	203436 - 204491	-	351	Hypothetical protein	*Vibrio* phage KVP40 (0.0)	99.4	0	N
	303	204569 - 205264	+	231	Hypothetical protein	*Vibrio* phage V09 (2e-172)	100	0	N
	304	205267 - 205659	+	130	Hypothetical protein	*Vibrio* phage KVP40 (5e-93)	100	0	N
	305	205664 - 205903	+	79	Hypothetical protein	*Vibrio* phage KVP40 (7e-49)	100	3	N
	306	205903 - 206235	+	110	Hypothetical protein	*Vibrio* phage KVP40 (6e-77)	100	0	N
	307	206235 - 206516	+	93	Hypothetical protein	*Vibrio* phage KVP40 (3e-63)	100	0	N
	308	206513 - 206746	+	77	Hypothetical protein	*Vibrio* phage KVP40 (3e-48)	100	1	N
	309	206743 - 206913	+	56	Hypothetical protein	*Vibrio* phage KVP40 (2e-33)	100	0	N
	310	206910 - 207098	+	62	Hypothetical protein	*Vibrio* phage KVP40 (9e-38)	100	0	N
	311	207128 - 207430	+	100	Hypothetical protein	*Vibrio* phage KVP40 (4e-68)	100	0	N
	312	207420 - 207704	+	94	Hypothetical protein	*Vibrio* phage KVP40 (1e-64)	100	0	N
	313	207708 - 208001	+	97	Hypothetical protein	*Vibrio* phage KVP40 (6e-65)	100	0	N
	314	207998 - 208918	+	306	Hypothetical protein	*Vibrio* phage VH1_2019 (0.0)	100	0	N
	315	208921 - 209772	+	283	Hypothetical protein	*Vibrio* phage phi-pp2 (0.0)	99.7	0	N
	316	209882 - 210181	+	99	Hypothetical protein	*Vibrio* phage KVP40 (1e-66)	100	0	N
	317	210242 - 210556	+	104	Hypothetical protein	*Vibrio* phage phi-pp2 (1e-70)	100	0	N
Metabolism	318	210556 - 211008	+	150	dCMP deaminase [IPR015517; dCMP_deaminase-rel; Deoxycytidylate deaminase-related]	*Vibrio* phage KVP40 (1e-109)	100	0	N

	319	211063 - 211989	+	308	NADPH-dependent 7-cyano-7-deazaguanine reductase [IPR007115; 6-PTP_synth/QueD; 6-pyruvoyl tetrahydropterin synthase/QueD family]	*Vibrio* phage KVP40 (0.0)	100	0	N
	320	212058 - 212726	+	222	GTP cyclohydrolase I [IPR001474; GTP_CycHdrlase_I; GTP cyclohydrolase I]	*Vibrio* phage KVP40 (7e-166)	100	0	N
	321	212730 - 214106	+	458	Hypothetical protein [IPR036511; TGT-like_sf; Queuine tRNA-ribosyltransferase-like]	*Vibrio* phage KVP40 (0.0)	100	0	N
	322	214136 - 215044	+	302	NADPH-dependent 7-cyano-7-deazaguanine reductase [IPR016428; QueF_type2; NADPH-dependent 7-cyano-7-deazaguanine reductase, QueF type 2]	*Vibrio* phage KVP40 (0.0)	100	0	N
	323	215100 - 215816	+	238	7-cyano-7-deazaguanine synthase [IPR018317; QueC; Queuosine biosynthesis protein QueC]	*Vibrio* phage KVP40 (3e-178)	100	0	N
	324	215816 - 216424	+	202	Hypothetical protein [IPR017932; GATase_2_dom; Glutamine amidotransferase type 2 domain]	*Vibrio* phage KVP40 (3e-151)	100	0	N
	325	216433 - 216708	+	91	Hypothetical protein [IPR024401; WYL_prot; WYL domain containing protein]	*Vibrio* phage KVP40 (2e-60)	100	0	N
	326	216701 - 216901	+	66	Hypothetical protein	*Vibrio* phage KVP40 (4e-42)	100	0	N
	327	217007 - 217213	+	68	Hypothetical protein	*Vibrio* phage phi-pp2 (8e-43)	100	0	N
Structure	328	217213 - 217551	+	112	Head assembly chaperone protein [IPR016416; Phage_T4_Gp31_GroEL; Bacteriophage T4, Gp31, chaperonin-GroEL]	*Vibrio* phage KVP40 (6e-75)	100	0	N
	329	217553 - 217942	+	129	Hypothetical protein	*Vibrio* phage KVP40 (3e-89)	100	0	N
Metabolism	330	217999 - 218508	-	169	Recombination endonuclease VII [IPR004211; Endonuclease_7; Recombination endonuclease VII]	*Vibrio* phage phi-pp2 (4e-124)	100	0	N
	331	218588 - 218842	+	84	Hypothetical protein	*Vibrio* phage phi-pp2 (3e-54)	100	0	N
Metabolism	332	218852 - 219859	+	335	RNA ligase [IPR012647; RNA_lig_RNL2; RNA ligase, Rnl2]	*Vibrio* phage phi-pp2 (0.0)	100	0	N
	333	219862 - 220056	+	64	Hypothetical protein	*Vibrio* phage KVP40 (1e-37)	100	2	N
	334	220084 - 220632	+	182	Hypothetical protein	*Vibrio* phage VH1_2019 (6e-134)	100	0	N
	335	220794 - 221321	+	175	Hypothetical protein	*Vibrio* phage KVP40 (8e-126)	100	0	N
Metabolism	336	221324 - 221902	+	192	DNA methyltransferase [IPR029063; SAM-dependent_MTases_sf; S-adenosyl-L-methionine-dependent methyltransferase superfamily]	*Vibrio* phage phi-pp2 (3e-144)	100	0	N
	337	221899 - 222108	+	69	Hypothetical protein	*Vibrio* phage phi-pp2 (2e-43)	100	0	N
	338	222129 - 222482	+	117	Hypothetical protein	*Vibrio* phage KVP40 (3e-75)	100	0	N
	339	222479 - 222751	+	90	Hypothetical protein	*Vibrio* phage phi-pp2 (1e-60)	100	0	N
	340	222744 - 223046	+	100	Hypothetical protein	*Vibrio* phage V09 (9e-69)	100	0	N
	341	223102 - 223239	+	45	Hypothetical protein	*Vibrio* phage phi-pp2 (5e-22)	100	0	N
Lysis	342	223247 - 225316	+	689	RIIA lysis inhibitor	*Vibrio* phage V09 (0.0)	100	0	N
	343	225309 - 226346	+	345	RIIB lysis inhibitor	*Vibrio* phage V09 (0.0)	100	0	N
	344	226426 - 226740	+	104	Hypothetical protein	*Vibrio* phage VH1_2019 (5e-71)	100	0	N
	345	226746 - 227432	+	228	Hypothetical protein [IPR013099; K_chnl_dom; Potassium channel domain]	*Vibrio* phage V09 (5e-167)	100	2	N
	346	227429 - 227938	+	169	Hypothetical protein	*Vibrio* phage V09 (6e-116)	99.4	0	Y
	347	227965 - 228507	-	180	Hypothetical protein	*Vibrio* phage V09 (2e-126)	100	0	N
	348	228768 - 229184	+	138	Hypothetical protein	*Vibrio* phage KVP40 (3e-96)	100	0	N
	349	229181 - 229456	+	91	Hypothetical protein	*Vibrio* phage KVP40 (2e-62)	100	0	N
Metabolism	350	229453 - 230493	+	346	Nucleotidyltransferase [IPR018775; RlaP; RNA repair pathway DNA polymerase beta]	*Vibrio* phage KVP40 (0.0)	100	0	N

	351	230528 - 230779	+	83	Hypothetical protein	*Vibrio* phage KVP40 (1e-52)	97.6	0	N
	352	230837 - 231211	+	124	Hypothetical protein [IPR019627; YAcAr; YspA, cpYpsA-related SLOG family]	*Vibrio* phage V09 (1e-87)	100	0	N
Metabolism	353	231223 - 232488	+	421	DNA helicase Dda [IPR041214; SH3_14; Dda helicase SH3 domain]	*Vibrio* phage phi-pp2 (8e-43)	100	0	N
	354	232567 - 232896	+	109	Hypothetical protein	*Vibrio* phage KVP40 (1e-76)	100	1	N
	355	232893 - 233072	+	59	Hypothetical protein	*Vibrio* phage KVP40 (1e-36)	100	0	N
	356	233140 - 233901	+	253	Hypothetical protein	*Vibrio* phage KVP40 (0.0)	100	0	N
	357	233965 - 234432	+	155	Hypothetical protein	*Vibrio* phage KVP40 (2e-111)	100	0	Y
	358	234437 - 234955	+	172	Hypothetical protein	*Vibrio* phage KVP40 (2e-124)	100	0	N
Metabolism	359	235030 - 236055	+	341	Nicotinamide-nucleotide adenylyltransferase NadM family/ADP-ribose pyrophosphatase [IPR000086; NUDIX_hydrolase_dom; NUDIX hydrolase domain]	*Vibrio* phage KVP40 (0.0)	100	0	N
	360	236090 - 236365	+	91	Hypothetical protein	*Vibrio* phage phi-pp2 (6e-60)	100	0	N
Metabolism	361	236362 - 236988	+	208	Nicotinate-nucleotide adenylyltransferase	*Vibrio* phage KVP40 (9e-153)	100	0	N
	362	236985 - 237170	+	61	Hypothetical protein	*Vibrio* phage KVP40 (8e-36)	100	0	N
	363	237167 - 237376	+	69	Hypothetical protein	*Vibrio* phage phi-pp2 (1e-42)	100	0	N
	364	237373 - 237579	+	68	Hypothetical protein	*Vibrio* phage KVP40 (8e-42)	100	0	N
	365	237576 - 237809	+	77	Hypothetical protein	*Vibrio* phage phi-pp2 (8e-49)	100	0	N
	366	237806 - 237979	+	57	Hypothetical protein	*Vibrio* phage PVA8 (4e-33)	100	0	N
	367	237976 - 238305	+	109	Hypothetical protein	*Vibrio* phage V09 (2e-74)	99.1	0	N
	368	238383 - 238574	+	63	Hypothetical protein	*Vibrio* phage phi-pp2 (4e-35)	100	2	N
	369	238578 - 238733	+	51	Hypothetical protein	*Vibrio* phage V09 (2e-22)	90.2	2	N
	370	238730 - 239062	+	110	Hypothetical protein	*Vibrio* phage KVP40 (2e-72)	93.7	1	N
	371	239066 - 239233	+	55	Hypothetical protein	*Vibrio* phage V09 (4e-28)	96.4	2	N
	372	239655 - 239777	+	40	Hypothetical protein	*Vibrio* phage phi-pp2 (8e-22)	100	1	N
	373	239777 - 240085	+	102	Hypothetical protein	*Vibrio* phage phi-pp2 (1e-69)	99	0	N
	374	240095 - 240700	+	201	Hypothetical protein	*Vibrio* phage PVA8 (2e-33)	98.4	0	N
	375	240784 - 241140	+	118	Hypothetical protein	*Vibrio* phage KVP40 (1e-83)	100	0	N
	376	241220 - 241501	+	93	Hypothetical protein	*Vibrio* phage PG216 (2e-61)	100	0	N
	377	241525 - 241914	+	129	Hypothetical protein	*Vibrio* phage V09 (1e-89)	100	0	N
	378	241991 - 242239	+	82	Hypothetical protein	*Vibrio* phage phi-pp2 (9e-52)	98.8	0	N
	379	242251 - 242664	+	137	Hypothetical protein	*Vibrio* phage KVP40 (2e-97)	100	0	N
	380	242667 - 243014	+	115	Hypothetical protein	*Vibrio* phage V09 (2e-80)	100	0	N
	381	243011 - 243259	+	82	Hypothetical protein	*Vibrio* phage phi-pp2 (3e-53)	100	1	N
	382	243244 - 243555	+	103	Hypothetical protein	*Vibrio* phage KVP40 (3e-69)	100	0	N

Table S2. Quantification of the AHPND toxin gene *pirA* in shrimp hepatopancreatic tissues by quantitative PCR. Hepatopancreatic tissues were collected from dead shrimp at day 2 and surviving shrimp at day 4 following challenge with *Vp_{AHPND}* strain 13-028/A3 and phage vB_VpM-pA3B5. *Ct* values and estimated *pirA* gene copy numbers are presented for the A3, A3/phage, and phage groups. ND, not detected.

Group	Time (day)	*Ct* value	Copies/ul
A3	2	24.90 ± 0.19	$3.15 \times 10^6 – 4.33\times10^6$
	4	31.48 ± 0.74	$4.14 \times 10^3 – 1.42 \times 10^4$
A3/phage	2	27.46 ± 0.47	$2.15 \times 10^5 – 4.90 \times 10^5$
	4	31.62 ± 0.38	$5.65 \times 10^3 – 1.12\times 10^4$
Phage	2	ND	-
	4		
